# EGFR‐TKIs Induced DPP4 Drives Metabolic Reprogramming of Persister Cells in Lung Cancer

**DOI:** 10.1002/advs.202506950

**Published:** 2025-06-06

**Authors:** Yuanzhou Zhang, Xiaojun Zhang, Xupeng Yang, Xingshi Chen, Yuehong Wang, Jingying Hu, Rui Liu, Xiaoying Luo

**Affiliations:** ^1^ State Key Laboratory of Systems Medicine for Cancer Shanghai Cancer Institute Renji Hospital Shanghai Jiao Tong University School of Medicine Shanghai China; ^2^ Department of Liver Surgery and Transplantation and Key Laboratory of Carcinogenesis and Cancer Invasion (Ministry of Education) Liver Cancer Institute Zhongshan Hospital Fudan University Shanghai China; ^3^ Department of Thoracic Surgical Oncology Shanghai Lung Cancer Center Shanghai Chest Hospital Shanghai Jiao Tong University School of Medicine Shanghai China

**Keywords:** DTP, DPP4, EGFR‐Tki, lung cancer, OXPHOS

## Abstract

Mutations in epidermal growth factor receptor (EGFR) are the key drivers of lung cancer initiation and recurrence. The cancer cells undergo transformation to a reversible drug‐tolerant persister (DTP) state prior to the development of resistance against EGFR‐tyrosine kinase inhibitors (TKIs). Two DTP lung cancer cells with different proliferative capacities are established and identified dipeptidyl peptidase 4 (DPP4) as a potential therapeutic target. The DTP cells primarily relied on oxidative phosphorylation, which is accompanied by the up‐regulation of fatty acid metabolism. Mechanistically, DPP4 facilitates the uptake of fatty acids via carnitine palmitoyl transferase 1a (CPT1A, and enhances fatty acid oxidation. In addition, the DPP4‐mitogen‐activated protein kinase kinase (MEK)‐Nuclear factor erythroid‐2‐related factor 2 (Nrf2) signaling pathway maintains mitochondrial function by activating the antioxidant pathway. The combination of osimertinib and sitagliptin, a DPP4 inhibitor, not only suppressed tumor progression but also reduced the number of residual tumor cells and minimal residual disease. Notably, this combination therapy significantly lowered recurrence rates and extended the survival of tumor‐bearing mice compared to the monotherapies. The study provides new insights into the metabolic adaptations of DTP lung cancer cells in response to EGFR‐TKIs, offering novel therapeutic strategies for targeting these persister cells.

## Graphical abstract

Drug‐tolerant persister (DTP) cells emerge early during EGFR‐TKI treatment, preceding the development of acquired resistance. Elevated DPP4 in DTP cells drives metabolic reprogramming and antioxidant adaptation, promoting cell survival under drug pressure. Pharmacological inhibition of DPP4 with sitagliptin disrupts this protective state, enabling effective elimination of residual cells before stable resistance is established.

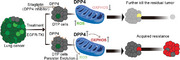

## 1 Introduction

Mutations in epidermal growth factor receptor (EGFR) are critical drivers of lung cancer, positioning EGFR‐tyrosine kinase inhibitors (TKIs) as the cornerstone of targeted therapy.^[^ ^1^ ^]^ EGFR‐TKIs have been recommended as the first‐line treatment for EGFR‐mutant lung cancer by the American Society of Clinical Oncology, the European Society for Medical Oncology, and the Chinese Society of Clinical Oncology.^[^ ^2^ ^]^ However, the tumor cells eventually develop resistance against the TKIs, leading to disease progression and recurrences.^[^ ^3^ ^]^ There are currently no well‐defined strategies that can overcome the mechanisms of acquired resistance to EGFR‐TKIs.

Drug‐tolerant persister (DTP) cells emerge early during chemotherapy, and have a reversible and tolerant phenotype that is distinct from conventional acquired resistance.^[^ ^4^ ^]^ DTP cells can potentially evolve into the truly drug‐resistant phenotype, and escape the selective pressure of EGFR‐TKIs.^[^ ^5^ ^]^ While the typical DTP cells exist in a “quiescent” state, the recently identified cycling DTP (c‐DTP) cells^[^ ^6^ ^]^ can re‐enter the cell cycle and continue to proliferate under drug exposure, thus contributing to minimal residual disease (MRD) in response to targeted therapies.^[^ ^7^ ^]^ Both DTP cells and c‐DTP cells could contribute to drug resistance and tumor recurrence. Due to their early emergence, and the lack of drug resistance and gene mutations, DTP cells are attractive therapeutic targets.^[^ ^4^, ^8^ ^]^ Furthermore, DTP cells exhibit a distinct metabolic pattern compared to typical tumor cells, which is driven by the environmental changes induced by drug exposure.^[^ ^9^ ^]^ DTP cells rely more heavily on mitochondrial respiration for energy and survival, in contrast to the parental cancer cells that generally depend on enhanced anaerobic glycolysis and lactate production.^[^ ^10^ ^]^ In B‐Raf proto‐oncogene (BRAF)‐mutant melanomas, post‐treatment persister cells express higher levels of mitochondrial oxidative‐Adenosine triphosphate (ATP) synthase and are susceptible to the inhibitors of mitochondrial respiration.^[^ ^11^ ^]^ Lung adenocarcinoma cells enter a static DTP state and activate PTEN‐induced putative kinase 1 (PINK1)‐mediated mitochondrial autophagy to promote oxidative phosphorylation, which allows them to survive treatment with mitogen‐activated protein kinases (MAPK) inhibitors.^[^ ^10a^ ^]^ Moreover, persistent cancer cells actively engage in fatty acid beta‐oxidation (FAO), a critical energy‐producing pathway in the mitochondrial respiratory chain.^[^ ^6^, ^12^ ^]^ Therefore, understanding the mechanism underlying the survival of DTP cells can aid in their selective elimination, thereby postponing recurrence, and improving the efficacy of EGFR‐TKIs.^[^ ^13^ ^]^

In this study, we treated the PC9 cells at Emax concentration of treatment. And then we isolated the DTP and c‐DTP subsets based on the EGFR ‐TKIs (gefitinib or osimertinib) and used live‐cell imaging to quantify the number and timing of division events over the course of proliferation rates and half‐maximal inhibitory concentrations (IC50) of the drugs in each residual single cell clones. Analysis of the putative cell surface targets of the drug‐treated DTP and c‐DTP cells revealed over‐expression of DPP4. However, no change in DPP4 was observed in the resistant cells, indicating that the upregulation of DPP4 is an early event in the evolution of resistance to EGFR‐TKIs. Moreover, DPP4 levels were markedly increased in the subcutaneous tumor tissues post‐treatment, which simulated the minimal residual disease (MRD), as well as in the orthotopic tumors receiving osimertinib and residual lesions from patients after EGFR‐TKIs treatment.

The DTP and c‐DTP cells underwent metabolic reprogramming at 90% of the inhibitory concentrations (IC_90_) of EGFR‐TKIs through the DPP4‐related signaling network. DPP4 enhanced fatty acid uptake, augmented FAO, and enabled the metabolic switch from glycolysis to oxidative phosphorylation (OXPHOS) in these cells by upregulating the mitochondrial transporter protein carnitine palmitoyl transferase 1a (CPT1A). Furthermore, high expression levels of DPP4 in the DTP and c‐DTP cells activated the Nuclear factor erythroid‐2‐related factor 2 (Nrf2)‐mediated anti‐oxidative stress pathway. This not only lowered the intracellular levels of reactive oxygen species (ROS) induced by EGFR‐TKIs but also regulated the cell cycle, which may be critical to evade the cytotoxic effects of the drugs. In addition, lower ROS production also maintains mitochondrial activity, which is essential for fatty acid transport and OXPHOS. Sitagliptin, a small molecule inhibitor of DPP4, reduced fatty acid uptake, FAO, and the protein levels of DPP4 and its downstream effectors in the persister cells in vitro, which decreased the number of residual cells. In addition, sitagliptin also limited tumor growth and led to the shrinkage of MRD tissues in vivo when combined with EGFR‐TKIs. Furthermore, targeted inhibition of DPP4 delayed tumor recurrence in combination with EGFR‐TKIs. Thus, disrupting the sustained metabolic reprogramming in DTP cells may offer a strategy to mitigate acquired resistance to EGFR‐TKIs and prevent tumor recurrence in lung cancer patients.

## 2 Results

### 2.1 DPP4 was Up‐Regulated in Persister Cells Induced by EGFR‐TKIs

**Figure 1:**
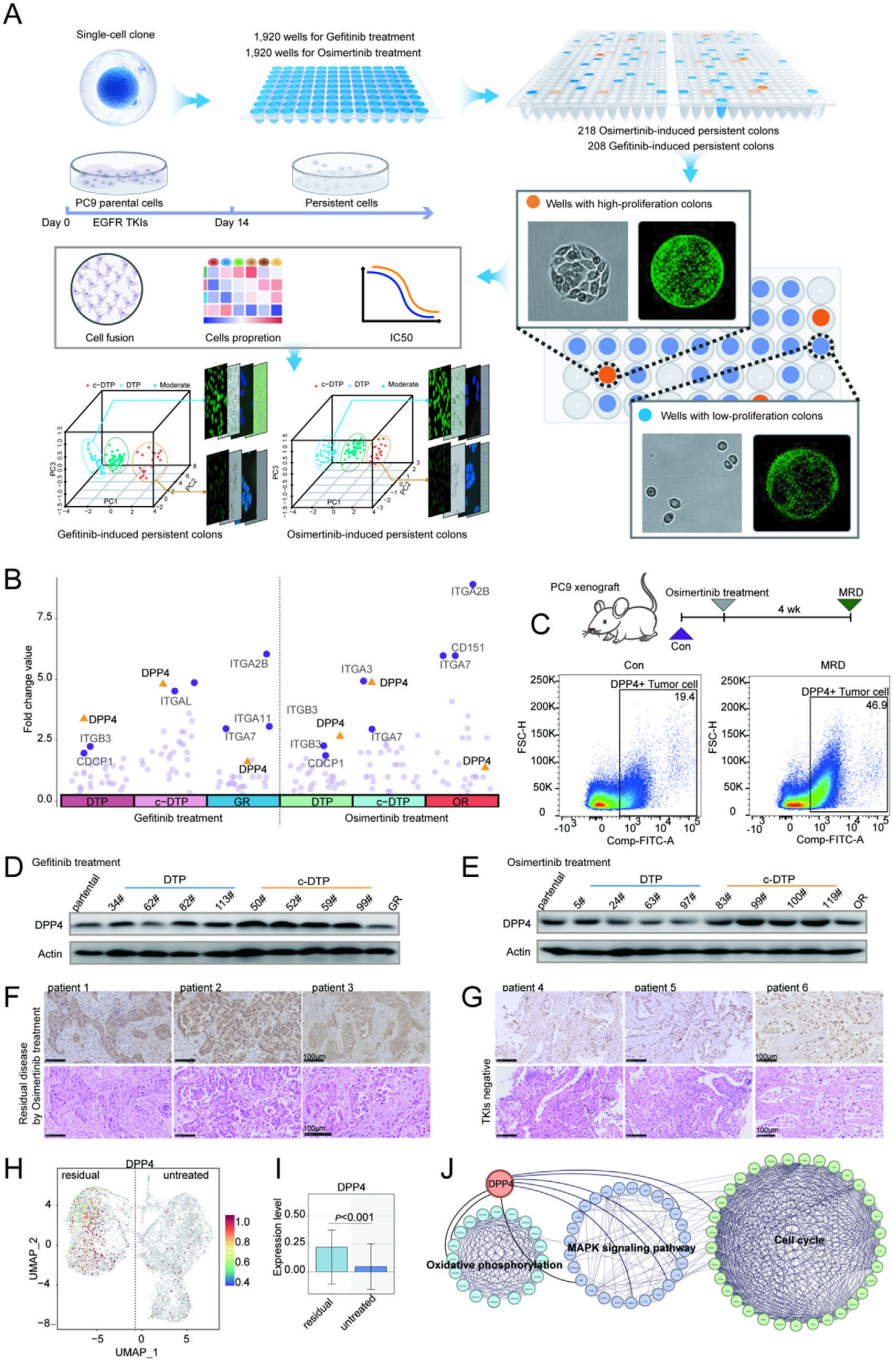
DPP4 was up‐regulated in persister cells induced by EGFR‐TKIs. A) Schematic diagram of drug‐resistant persistent cells construction, cluster analysis and clones subsets identification (c‐DTP cells with high proliferation and DTP cells with low proliferation). Single cell clone of PC9 cells was treated with 300 µM osimertinib or 500 µM gefitinib for 14 days to obtain surviving single cell clone. The proliferation ability and IC50 of these surviving cells were monitored in real time, and cells population analysis was carried out appropriately. Four representative DTP clones and four c‐DTP clones were screened out and the aging markers, cell cycle and proliferation ability were identified respectively. B) The mRNA expression of 36 cell surface markers in parental cells, DTP cells, c‐DTP cells and osimertinib /gefitinib‐resistant cells was quantitatively determined by RT‐qPCR. Fold change values are calculated relative to parental cells; *n* =  3, Paired two‐tailed Student's t‐test. C) Scheme and flow cytometry plot of DPP4‐positive cells in subcutaneous xenograft tumors; *n* =  5 xenograft tumors. D,E) Western blot analysis of DPP4 in parental cells, DTP cells, c‐DTP cells, and drug‐resistant cells treated with osimertinib or gefitinib. F,G) Representative immunohistochemistry against DPP4 gene in residual tumors by Osimertinib treatment and in EGFR TKIs negative tumors, Scale bars, 100 µM. H) Feature plot depicting expression of DPP4 in individual cells. I) Box plot showing the expression levels of DPP4 in Residual and Untreated groups, with differences tested using the Wilcoxon test. J) Analysis of DPP4‐mediated interaction networks by transcriptome sequencing of drug‐resistant persistent cells.

Determining the mechanisms by which PC9 cells survive and regain their proliferative capacity under EGFR‐TKI treatment necessitates a detailed analysis of the cellular and molecular characteristics of these rare cell populations during the initial phases of therapy. To this end, we obtained DTP and c‐DTP clones of PC9 cells by treating them with E_max_ concentrations of gefitinib (500 µM) or osimertinib (300 µM) for 14 days, which resulted in the expected small population of viable and quiescent DTP cells. The proliferative capacity of the surviving persister cell clones, and the IC_50_ of the drugs in each single clone were determined by live‐cell imaging. We then further isolated highly proliferative c‐DTP cells (orange) and less proliferative DTP cells (blue) based on their proliferative capacity (**Figure** **1**A; Figure S1A–D, Supporting Information). We selected four independent DTP and c‐DTP clones and validated their specificity, including markers of aging (β‐galactosidase, Figure S1E,F, Supporting Information), cell cycle (Figure S2A,B, Supporting Information), proliferative capacity (Figure S2C,D, Supporting Information), growth recovery under drug douche (Figure S3A–D, Supporting Information) and aldehyde dehydrogenase (ALDH) activity^[^ ^14^ ^]^ (Figure S20, Supporting Information). ALDH is higher in both subtypes of DTP cells than in parental cells, and ALDH levels affect ROS, DNA‐protein adducts, and p53 signaling, all of which can lead to more aggressive, recurrent, and drug‐resistant cancers.^[^ ^15^ ^]^ The DTP cells exhibited an aging phenotype with slow proliferation, G1 phase arrest of the cell cycle, and increased cellular oxidative stress levels. In contrast, the c‐DTP cells showed a non‐senescent phenotype.

We analyzed the expression of 36 major cancer cell surface markers in the parental cells, DTP cells, c‐DTP cells, and drug‐resistant cells (Figure 1B; Figure S5A–F, Supporting Information). DPP4 mRNA levels increased by threefold or more in the DTP and c‐DTP cells compared to parental cells, whereas no significant changes were observed in the drug‐resistant cells. Once mice developed subcutaneous xenograft tumors, we treated them with osimertinib until the rate of tumor regression plateaued, indicating MRD. As shown in Figure 1C, the positive rate of DPP4 in the tumor cells of MRD after osimertinib treatment was much higher than that of xenograft tumors before treatment. In addition, DPP4 protein expression was also upregulated in DTP and c‐DTP cells compared to the parental cells and drug‐resistant cells (Figure 1D–E).

We also examined DPP4 expression in tumor tissues from lung cancer patients by immunohistochemistry, and found that DPP4 was significantly upregulated in the residual tumor tissues after osimertinib treatment compared to the tumor tissues from patients who did not receive EGFR‐TKIs (Figure 1F–G). Analysis of the single‐cell dataset of osimertinib‐treated lung cancer patients also indicated higher expression of DPP4 in the residual cancer cells (Figure 1H–I; Figure S4G–L, Supporting Information). Transcriptomic sequencing of the DTP and c‐DTP cells further revealed that DPP4 is central to a key signaling network that includes the cell cycle, OXPHOS, and MAPK/NRF2 signaling pathways, underscoring its critical role in the survival of persister cells (Figure 1I).

### 2.2 Oxidative Phosphorylation in Persister Cells Increased

**Figure 2:**
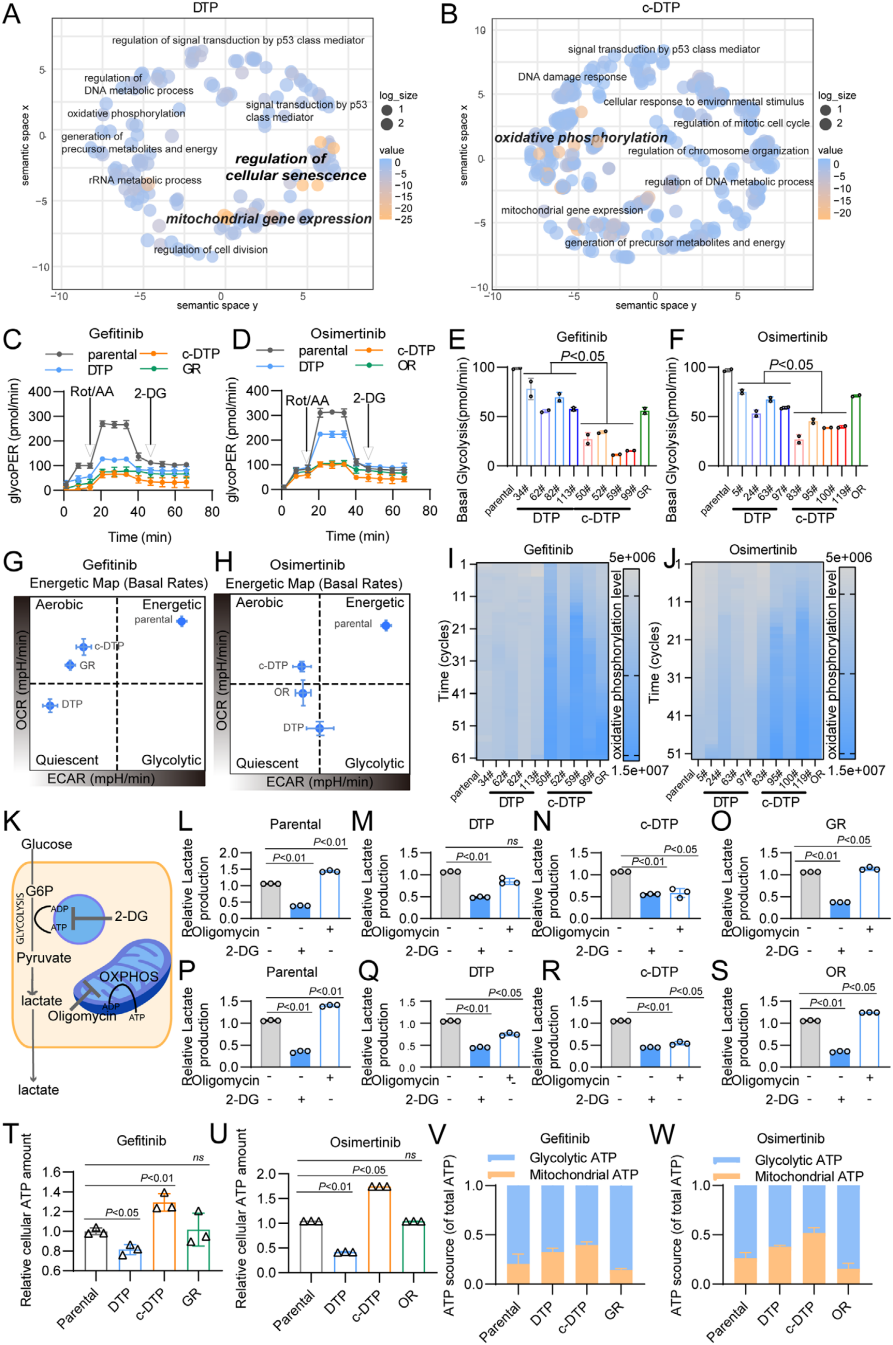
Oxidative phosphorylation in persister cells increased. A,B) Significantly enriched GO terms in biological process category in DTP cells and c‐DTP cells. The enrichment value is represented by the color intensity. The p‐value is represented by the circle size. C‐F) Glycolytic capacity of parental cells, DTP cells, c‐DTP cells and osimertinib/gefitinib‐resistant cells was measured using the Seahorse assay. Data are represented as mean ± SEM. Statistical differences determined with one way ANOVA and Tukey's multiple comparisons test. G,H) Metabolic phenotypes of parental cells, DTP cells, c‐DTP cells, and osimertinib/gefitinib‐resistant cells were assessed using the Seahorse assay. Data are represented as mean ± SEM. I,J) The oxidative phosphorylation level of parental cells, DTP cells, c‐DTP cells, and osimertinib/gefitinib‐resistant cells was detected by the oxidative phosphorylation probe kit. K‐S) According to the lactate production kit, Lactate levels of parental cells, DTP cells, c‐DTP cells, and osimertinib/gefitinib‐resistant cells were detected for absorbance; *n* =  3, Paired two‐tailed Student's t‐test. Data represented as mean ± SEM. T and U) According to the ATP production kit, the ATP production of parental cells, DTP cells, c‐DTP cells, and drug‐resistant cells was detected for absorbance. *n* =  3, Paired two‐tailed Student's t‐test. Data are represented as mean ± SEM. V and W) According to the ATP dependency kit, the dependence of ATP production on glycolysis or oxidative phosphorylation of parental cells, DTP cells, c‐DTP cells, and osimertinib/gefitinib‐resistant cells was detected for absorbance. *n* =  3, Paired two‐tailed Student's t‐test. Data are represented as mean ± SEM.

RNA sequencing (RNA‐seq) analysis of the DTP cells and c‐DTP cells showed the enrichment of distinct biological processes. While both subsets were associated with mitochondrial function, DTP cells were primarily enriched in cellular senescence and mitochondrial gene expression (**Figure** **2**A), whereas c‐DTP cells are mainly enriched in OXPHOS (Figure 2B). Furthermore, glycolysis levels were considerably reduced in the DTP and c‐DTP cells compared to the parental and drug‐resistant cells, and a more pronounced decrease was observed in c‐DTP cells (Figure 2C–F). The primary metabolic pathway in the persister cells had shifted from glycolysis, which is predominant in the parental cells, to OXPHOS (Figure 2G,H). The levels of OXPHOS substrates were significantly increased in the DTP and c‐DTP cells compared to parental cells, and the increase was particularly notable in c‐DTP cells (Figure 2I,J).

To further evaluate the dependence of the DTP cells on these metabolic pathways, we treated them with specific inhibitors of OXPHOS (oligomycin) and glycolysis (2‐DG), and measured lactate production. Both DTP and c‐DTP cells exhibited higher dependence on OXPHOS relative to the parental or drug‐resistant cells (Figure 2K–S). Although ATP levels were lower in DTP cells and increased significantly in the c‐DTP cells compared to parental or drug‐resistant cells (Figure 2T,U), both persister subsets showed increased reliance on OXPHOS for ATP production (Figure 2V,W).

### 2.3 Persister Cells Enhance Fatty Acid Uptake

**Figure 3:**
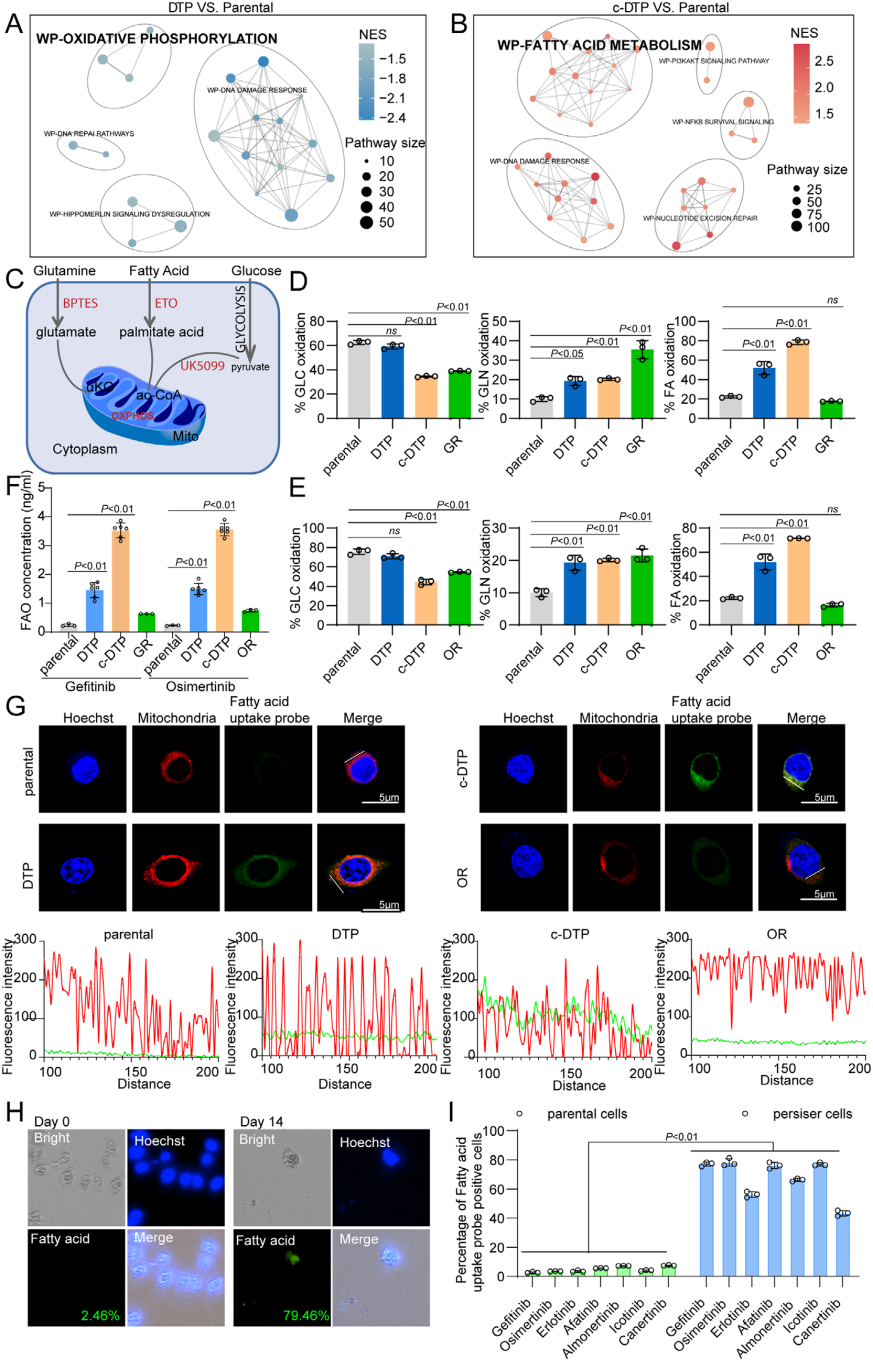
Persister cells enhance fatty acid uptake. A,B) Pathway network analysis of DTP cells and c‐DTP cells based on transcriptome sequencing data. The pathway size is represented by the circle size. Normalized Enrichment Score is represented by the color intensity. C‐E) Schematic diagram and basal mitochondrial respiration levels of parental cells, DTP cells, c‐DTP cells, and osimertinib/gefitinib‐resistant cells detecting by Seahorse assay; *n* =  4, Paired two‐tailed Student's t‐test. Data represented as mean ± SEM. F) According to the FAO kit, the FAO substrate of parental cells, DTP cells, c‐DTP cells, and osimertinib/gefitinib‐resistant cells was detected by enzyme‐labeled assay. *n* =  3, Paired two‐tailed Student's t‐test. Data are represented as mean ± SEM. G) Immunofluorescence images of nucleus (Hoechst33258, blue), mitochondria (red) and fatty acid uptake probe (green) in parental cells, DTP cells, c‐DTP cells, and osimertinib‐resistant cells. Scale bars, 5 µM. Data are representative of three independent experiments. H) Immunofluorescence images of fatty acid uptake in osimertinib‐induced persister cells detected by Celigo Image Cytometer. I) Fatty acid uptake was detected by fluorescent probe after treatment with different EGFR‐TKIs. Fluorescence results and single cell fluorescence intensity analysis were performed with Celigo Image Cytometer.

The metabolic network analysis of RNA‐seq data showed that the DTP and c‐DTP cells were mainly enriched in OXPHOS and fatty acid metabolism (**Figure** **3**A,B). We analyzed the oxidation levels of mitochondrial substrates using seahorse analysis, and observed increased reliance on fatty acids in the persister cells (Figure 3C–E). In fact, FAO was significantly higher in DTP cells and c‐DTP cells compared to parental cells or drug‐resistant cells (Figure 3F). To confirm these findings, we stained the DTP and c‐DTP cells with fluorescent probes for mitochondria (red) and fatty acids (green). The co‐localization of the signals indicated increased uptake of fatty acids into the mitochondria (Figure 3G; Figure S5, Supporting Information). We also observed substantially higher fatty acid uptake into the osimertinib‐treated persister cells using the Celigo Image Cytometer (Figure 3H). Additional DTP clones of PC9 cells were obtained by treating them with different EGFR‐TKIs (erlotinib, afatinib, almnertinib, icotinib, and cancertinib for 14 days (Figure 3I; Figure S6A,B, Supporting Information). Higher fatty acid uptake was consistent and widespread among these persister clones, which may play a critical role in their survival and metabolic adaptation.

### 2.4 DPP4‐CPT1A Axis Mediates Fatty Acid Uptake in Persister Cells

**Figure 4:**
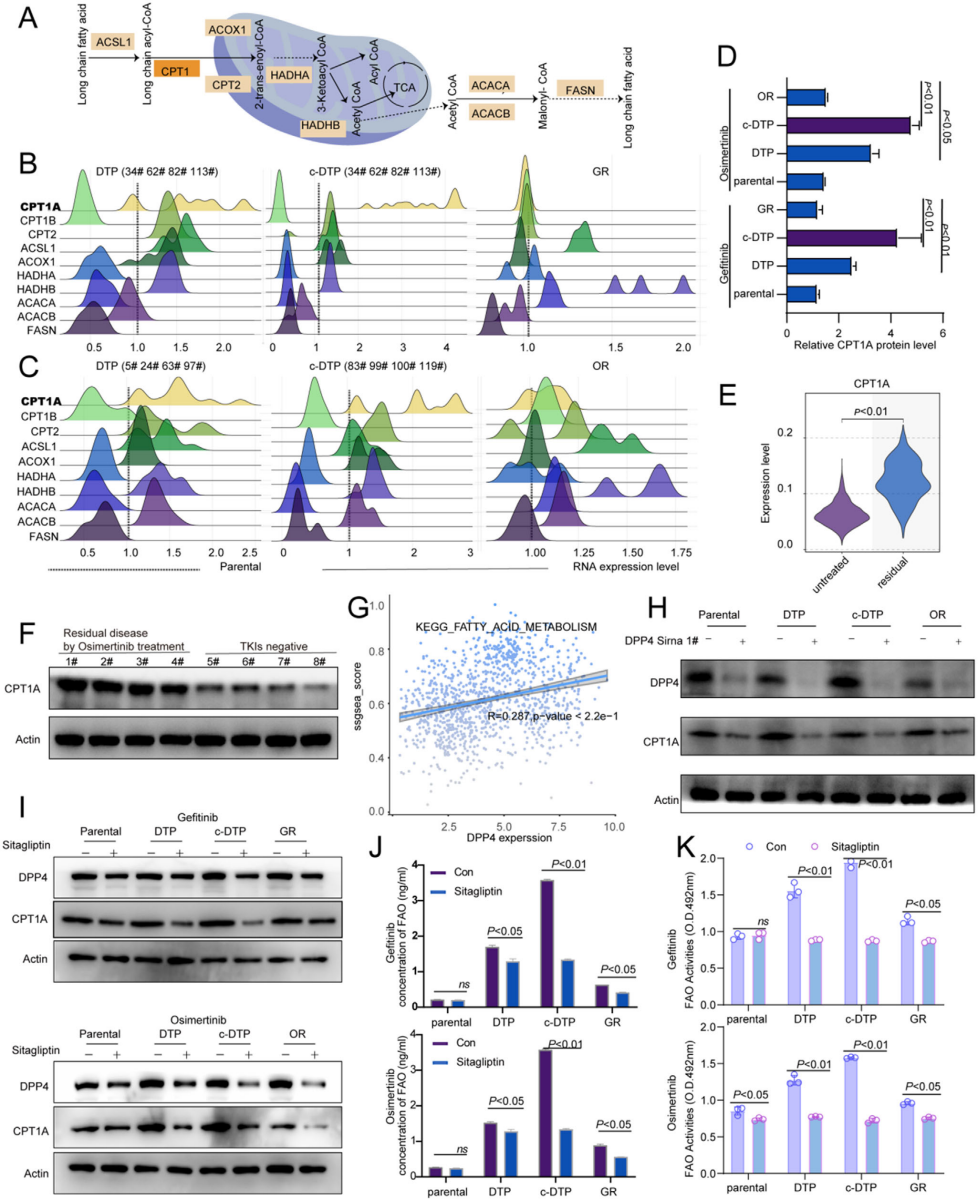
DPP4‐CPT1A axis mediates fatty acid uptake in persister cells. A‐C) Schematic diagram and quantitative RT‐qPCR analysis of 10 key enzymes involved in mitochondrial pathway in parental cells, DTP cells, c‐DTP cells, and osimertinib/gefitinib ‐resistant cell subsets were quantitatively determined by RT‐qPCR. Fold change values are calculated relative to parental cells; *n* =  3, Paired two‐tailed Student's t‐test. D) Western blot analysis of CPT1A in parental cells, DTP cells, c‐DTP cells, and osimertinib /gefitinib‐resistant cells. E) Single cell analysis of residual cells from sample_003 compared with untreated cells in CPT1A expression. F) Western blot analysis of CPT1A in residual tumors by Osimertinib treatment and EGFR TKIs negative tumors from patients. G) Spearman correlation analysis of DPP4 expression with fatty acid metabolic pathways using the KEGG dataset. H) Western blot analysis of DPP4 and CPT1A in parental cells, DTP cells, c‐DTP cells, and osimertinib‐resistant cells after DPP4 siRNA treatment. I) Western blot analysis of DPP4 and CPT1A in parental cells, DTP cells, c‐DTP cells, and osimertinib /gefitinib‐resistant cells after Sitagliptin treatment. J,K) According to the FAO kit, the FAO substrate and activities of parental cells, DTP cells, c‐DTP cells, and osimertinib/gefitinib‐resistant cells were detected for absorbance after Sitagliptin treatment; *n* =  3; Paired two‐tailed Student's t‐test. Data are represented as mean ± SEM.

**Figure 5:**
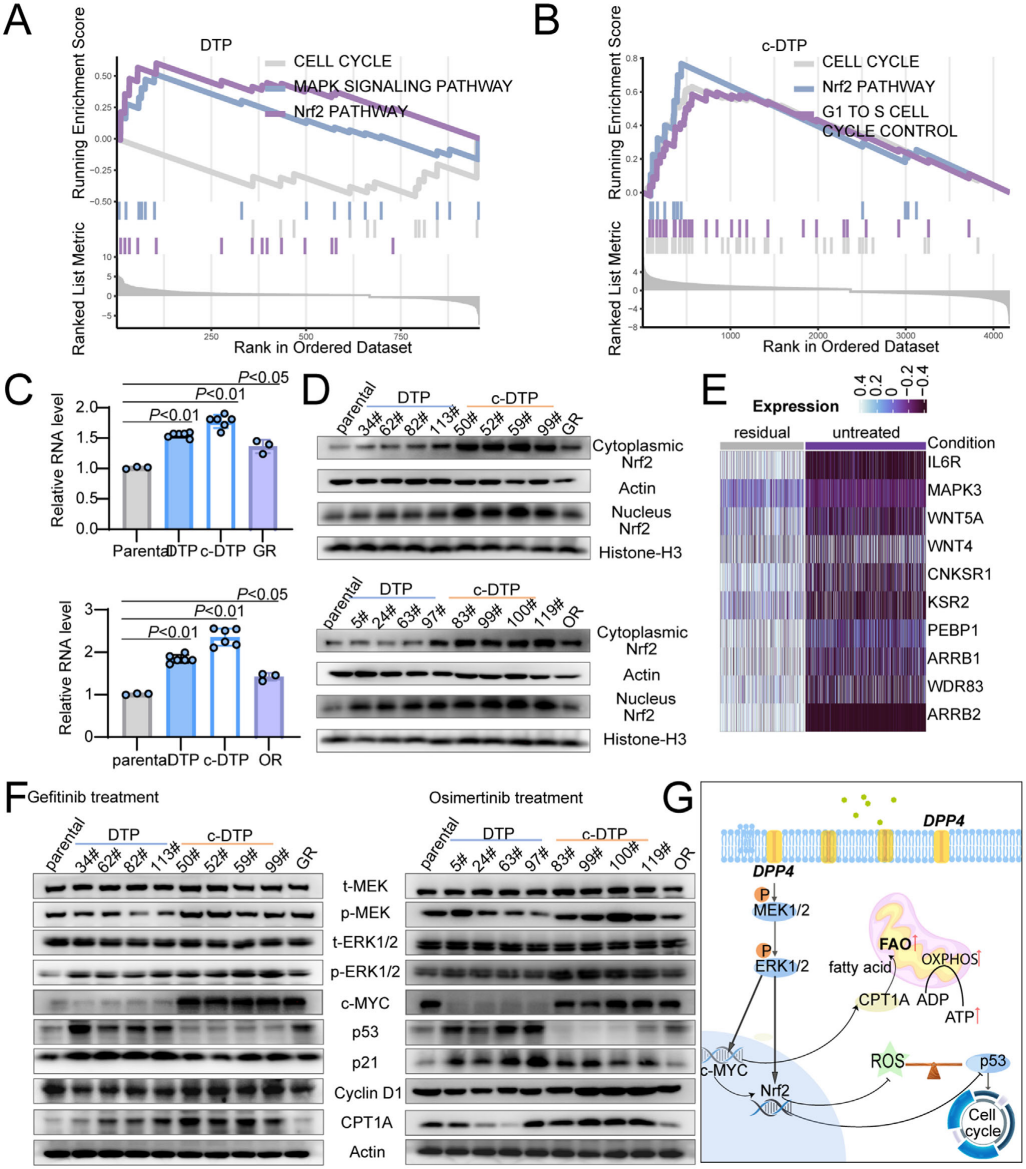
DPP4 regulates OXPHOS and cell cycle in the persister cells. A,B) Gene set enrichment analysis (GSEA) of transcriptome sequencing results of DTP cells and c‐DTP cells. C) Quantitative RT‐qPCR analysis of NRF2 expression in parental cells, DTP cells, c‐DTP cells and osimertinib/gefitinib‐resistant cells. Fold change values are calculated relative to parental cells; *n* =  3, Paired two‐tailed Student's t‐test. D) The NRF2 expression in the nucleus and cytoplasm of parental cells, DTP cells, c‐DTP cells, and osimertinib/gefitinib‐resistant cells was detected after isolating by nucleus extraction kit. Histone‐H3 is the internal reference of nuclear protein. E) Single cell heat map of MAPK gene set in sample_003. F) Western blot analysis of t‐MEK, p‐MEK, t‐ERK1/2, p‐ERK1/2, c‐MYC, p53, p21, Cyclin D1, and CPT1A in parental cells, DTP cells, c‐DTP cells, and osimertinib /gefitinib‐resistant cells. G) Schematic diagram of DPP4‐mediated signaling molecular networks regulating fatty acid uptake and cell cycle.

Based on the findings so far, we analyzed the expression levels of the key enzymes involved in mitochondrial fatty acid metabolism in the different clones. Carnitine palmitoyltransferase 1A (CPT1A) showed the most significant changes (**Figure** **4**A–C), and was upregulated in the DTP and c‐DTP cells (Figures 4D and **5**F). In addition, analysis of the single‐cell datasets showed that CPT1A expression was also significantly increased in residual cells following osimertinib treatment (Figure 4E). Consistent with this, CPT1A levels were elevated in the residual tumor tissues from lung cancer patients after osimertinib treatment (Figure 4F). Moreover, we identified a positive correlation between fatty acid metabolism and DPP4 expression in The Cancer Genome Atlas Lung Adenocarcinoma (TCGA‐LUAD) dataset (Figure 4G). To validate the above results, we knocked down DPP4 expression using specific siRNA (Figure S14A–D, Supporting Information), and observed a significant reduction in CPT1A mRNA and protein levels (Figure 4H; Figure S4E–H, Supporting Information).

Sitagliptin, as a small molecule inhibitor of DPP4, markedly lowered DPP4 and CPT1A protein levels (Figure 4I; Figure S13A, Supporting Information), and decreased FAO in the persister cells (Figure 4J,K). These findings strongly suggest that the DPP4‐CPT1A axis is crucial for the metabolic reprogramming and enhanced fatty acid uptake in persister cells, thus highlighting its potential as a therapeutic target.

### 2.5 DPP4 Regulates OXPHOS and Cell Cycle in the Persister Cells

We selected three representative DTP and c‐DTP clones along with their parental cells for transcriptome sequencing. Differential expression analysis and subsequent pathway enrichment analysis were performed by comparing DTP and c‐DTP clones individually against their respective parental cells to identify significant transcriptional alterations associated with the resistant phenotype. The Nrf2 pathway was highly enriched in the DTP and c‐DTP cells. On the other hand, the cell cycle pathway was upregulated in the c‐DTP cells and downregulated in the DTP cells (Figure 5A,B). Consistent with the above results, Nrf2 mRNA levels were significantly elevated in the DTP and c‐DTP cells (Figure 5C), and Nrf2 protein expression was elevated in the cytoplasm and nuclei of the persister cells (Figure 5D). Furthermore, the MAPK‐Nrf2 signaling pathway was upregulated in the single‐cell RNA‐seq data of samples from patients treated with osimertinib (Figure 5E; Figure S7A–H, Supporting Information), and that of DTP and c‐DTP cell clones (Figure S8A–I, Supporting Information). To investigate whether DPP4 upregulation drives the activation of Nrf2 and MAPK pathways in resistant cells, we performed correlation analyses using transcriptomic data. Results revealed strong positive correlations between DPP4 expression and both MAPK gene set expression (r = 0.952, p = 0.003; Figure S17A, Supporting Information) and Nrf2 expression (r = 0.969, p = 0.001; Figure S17B, Supporting Information). Notably, cells with higher DPP4 expression consistently showed elevated expression of individual MAPK pathway genes (Figure S17C, Supporting Information). These findings suggest that DPP4 upregulation is responsible for the activation of both Nrf2 and MAPK pathways in persister cells. The downstream signaling pathway of DPP4 was also activated in the DTP and c‐DTP cells, as indicated by the significant upregulation of t‐MEK, p‐MEK, t‐extracellular signal‐regulated kinase (ERK 1/2), P‐ERK1/2, and CPT1A. In addition, c‐MYC activation in these cells up‐regulated CPT1A expression (Figure 5F), which improved fatty acid uptake and sustained OXPHOS. Besides, analysis of the DPP4‐MAPK‐NRF2‐OXPHOS axis across cell groups demonstrated that c‐DTP cells maintained consistently high expression levels across all pathway components (Figure S17D, Supporting Information), while DTP cells showed notably lower expression levels. Parental cells exhibited minimal expression throughout the axis. This pattern further supports our hypothesis that DPP4 upregulation drives downstream MAPK and NRF2 pathway activation, ultimately enhancing OXPHOS in resistant cells.

Consistent with the results of mRNA sequencing, the cell cycle‐related proteins p53 and p21 were upregulated in the DTP cells and down‐regulated in c‐DTP cells (Figure 5F). On the other hand, cyclin D1 was down‐regulated in DTP cells and up‐regulated in c‐DTP cells. Nrf2 levels were considerably higher in the DTP and c‐DTP cells compared to the parental cells. However, the increase in Nrf2 expression was less pronounced in the DTP cells compared to the c‐DTP cells following exposure to EGFR‐TKIs. Consistent with this, the DTP cells exhibited higher levels of endogenous oxidative stress than the c‐DTP cells (Figures S9 and S10, Supporting Information). The elevated ROS levels in the DTP cells activated the p53 pathway (Figure 5G), resulting in cell cycle arrest and lower proliferation rates. On the other hand, the c‐DTP cells continued to proliferate even in the presence of EGFR‐TKIs. Additionally, the high ROS levels in DTP cells induced depolarization of the mitochondrial membrane, which may partially explain the lower levels of OXPHOS in DTP cells relative to c‐DTP cells (Figures S11 and S12, Supporting Information).

### 2.6 Sitagliptin and EGFR‐TKIs Synergistically Reduced Persister Cells and Residual Tumors

**Figure 6:**
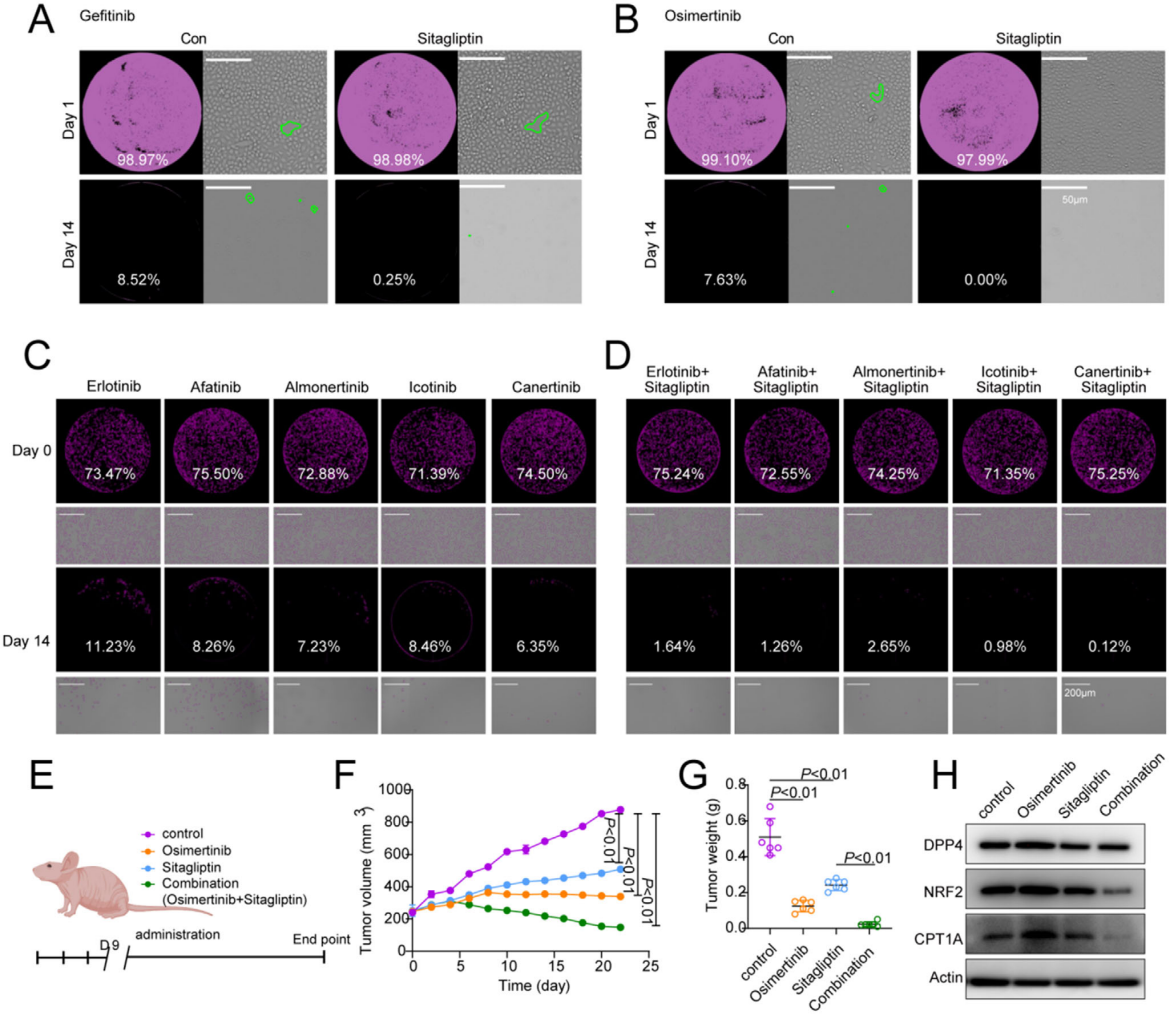
Sitagliptin and EGFR‐TKIs synergistically reduced persister cells and residual tumors. A and B) The number of cells after sitagliptin combined with gefitinib or osimertinib treatment was counted by Celigo Image Cytometer. Data are representative of three independent experiments. C,D) Cell survival rate in persister cells after multiple EGFR‐TKIs treatment combined with sitagliptin was detected by Celigo Image Cytometer. Representative cell images and analysis of PC9 cells treated with multiple EGFR‐TKIs in combination with sitagliptin were captured and analyzed. E) Treatment schematic diagram of constructing xenograft tumors with PC9 cells. F) PC9 subcutaneous xenograft growth; n =  6 xenografts per condition; mixed linear model with no adjustment for multiple comparisons. G) PC9 subcutaneous xenograft growth; n =  6 xenografts per condition; Paired two‐tailed Student's t‐test. Data are represented as mean ± SEM. H) Western blot analysis of DPP4, NRF2, and CPT1A in PC9 subcutaneous xenograft treated with osimertinib, sitagliptin, the combination or control.

The synergistic effect of sitagliptin and EGFR‐TKIs was evaluated by the BLISS method (Figure S13A–E, Supporting Information). We tracked the number of cells treated with Gefitinib and Osimertinib (**Figure** **6**A,B). After the addition of sitagliptin, the proportion of persistent cells almost disappeared. In addition, we extended the other six EGFR‐TKIs, and although the therapeutic effect of EGFR‐TKIs on PC9 cells varied, so did the proportion of persistent cells produced (close to 10%), the addition of sitagliptin further reduced the number of persistent cells, resulting in a synergistic effect (Figure 6C,D). We further validated the synergistic action of sitagliptin and osimertinib in vivo by establishing subcutaneous PC9 xenografts in mice (Figure 6E). The drug combination significantly inhibited tumor growth (Figure 6F) and reduced tumor volume (Figure 6G). Furthermore, DPP4 was elevated in residual tissues after osimertinib treatment, and the levels of DPP4, NRF2, and CPT1A subsequently decreased after sitagliptin treatment. And NRF2 and CPT1A were further reduced by the synergistic treatment of sitagliptin and osimertinib (Figure 6H; Figure S14I–L, Supporting Information). Peritumoral injection of sitagliptin did not affect blood glucose or body weight in mice (Figure S14K,L, Supporting Information) but significantly reduced the concentration and activity of FAO in tumor tissues Figure S14M,N, Supporting Information).

### 2.7 Sitagliptin Delayed Recurrence by Inhibiting Residual Tumors

**Figure 7:**
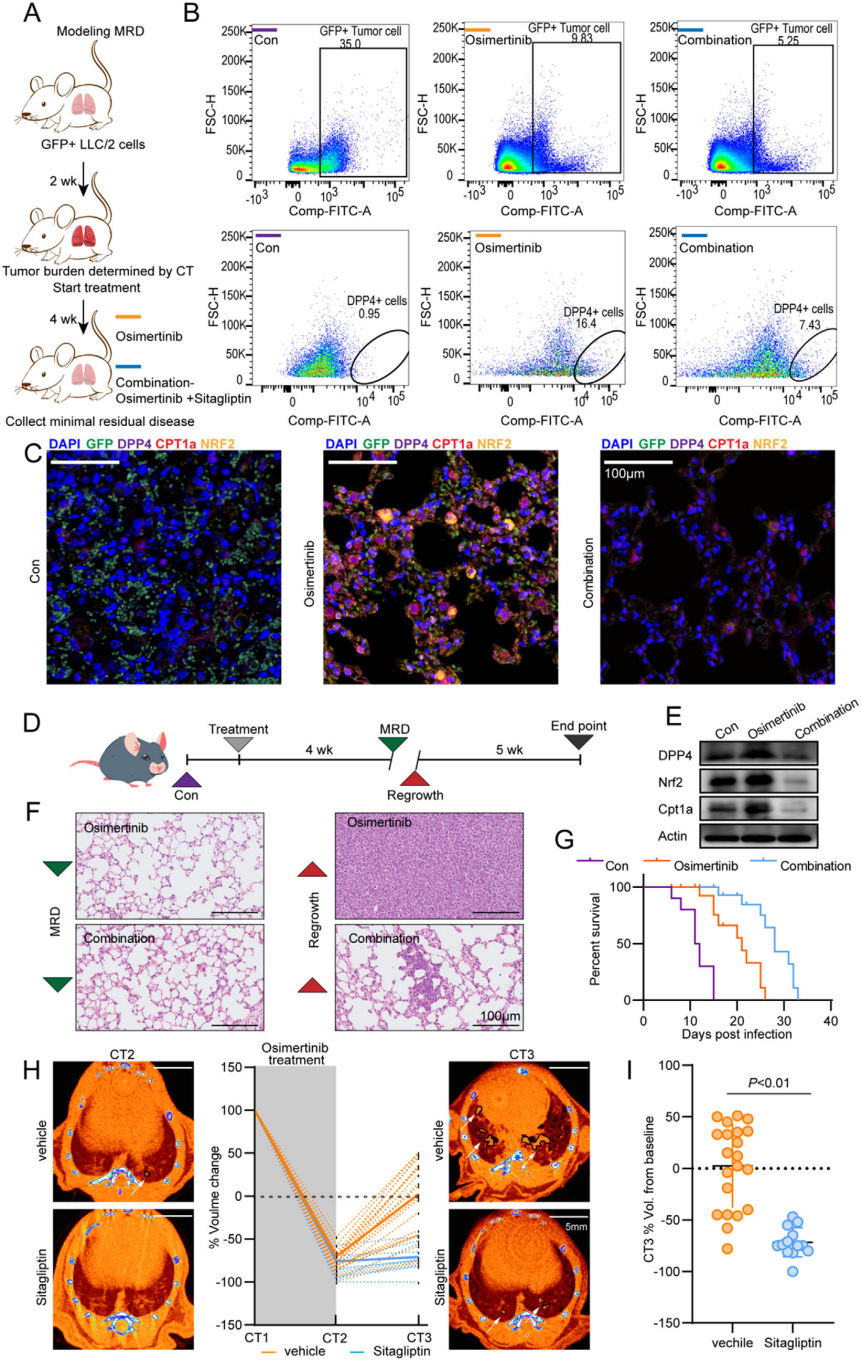
Sitagliptin delayed recurrence by inhibiting residual tumors. A) Schematic diagram of orthotopic lung cancer model and MRD model construction. B) Representative flow cytometric plots and statistical analysis of tumor cells (GFP+) proportion and positivity for DPP4 in lung tumor treated with osimertinib, the combination of sitagliptin and osimertinib or control.; *n* =  5 Lung tumors. C) Immunofluorescence images of DAPI, GFP, DPP4, NRF2, and CPT1A in lung tumor treated with osimertinib, the combination of sitagliptin and osimertinib or control. Scale:5 µm. D) Schematic diagram of the lung cancer MRD model and recurrence model. E) Western blot analysis of DPP4, NRF2 and CPT1A in lung tumor treated with osimertinib, the combination of sitagliptin and osimertinib or control. F) Representative H&E staining results of MRD model and recurrence model in lung tumor treated with osimertinib or combination. Data are representative of three independent experiments. G) Kaplan–Meier analysis of the experiment in D. A two‐tailed Wilcoxon test was used to compare differences in the average time to event. H) Representative CT scans (CT2 and CT3) of mice treated with control or sitagliptin after osimertinib treatment. The black line indicates the detected tumor. Orange area: bronchi and blood vessels. Scale:5 mm. Response of mice to sequential osimertinib‐sitagliptin treatment, expressed as percentage change in tumor volume relative to the start (left) and end point (right) of treatment. Sequential treatment with osimertinib (gray area) followed by sitagliptin. CT scans were performed every 1 month. I) Dotted lines represent individual tumors; Solid line represents the mean ± s.e.m. (left). Each dot represents a separate tumor; Black line indicates the average (right). P‐value calculated using two‐tailed Mann‐Whitney test.

Persister cells grow slowly and even enter quiescence in the presence of EGFR‐TKIs, and are responsible for MRD that eventually leads to recurrence.^[^ ^16^ ^]^ Therefore, we constructed an orthotopic lung cancer model using LLC/2 cells to monitor the efficacy of the combination treatment against recurrent tumor growth. While osimertinib monotherapy reduced the number of tumor cells in the MRD tissues, the number of DPP4‐positive tumor cells showed a significant increase. However, the combination of osimertinib and sitagliptin further reduced the overall tumor cell population and nearly halved the number of DPP4‐positive tumor cells compared to osimertinib alone (**Figure** **7**A,B; Figure S15A, Supporting Information). Additionally, sitagliptin treatment decreased FAO in the tumor tissues (Figure S15B,C, Supporting Information). Consistent with the above results, osimertinib monotherapy led to a dramatic increase in DPP4, Nrf2 and CPT1A expression in MRD tissues despite achieving a reduction in GFP‐positive tumor cells. The combination of osimertinib and sitagliptin resulted in a greater reduction in GPF‐positive tumor cells and a concurrent decrease in DPP4, Nrf2, and CPT1A expression (Figure 7C; Figure S16A–C, Supporting Information).

The mice bearing orthotopic tumors were treated with the different drugs for four weeks, and the treatment was ceased to restore tumor growth (Figure 7D). DPP4, NRF2, and CPT1A levels were elevated in the MRD tissues obtained from mice treated with osimertinib alone, whereas the combination of osimertinib and sitagliptin significantly downregulated these proteins (Figure 7E). As expected, the combination therapy also reduced the recurrence rate of residual tumors compared to osimertinib monotherapy (Figure 7F), which was accompanied by significantly improved survival outcomes (Figure 7G). Microcomputed tomography (µCT) showed that established tumors treated with osimertinib regressed effectively, and many nodules were undetectable after one month of treatment (Figure 7H,I). However, after halting osimertinib administration, the tumors that were presumably eliminated reappeared and nearly grew to pre‐treatment sizes within one month. In contrast, sitagliptin significantly inhibited tumor recurrence, and the size of the nodules was effectively controlled.

## 3 Discussion

Molecular targeted therapies have revolutionized cancer treatment in the last decades, notably with the advent of EGFR‐TKIs for lung cancer.^[^ ^17^ ^]^ Although targeted therapies have deepened our understanding of somatic genetic alterations, specifically “driver” mutations in oncogenic kinases,^[^ ^18^ ^]^ the success of these therapies is limited by the inevitable emergence of drug resistance, which ultimately leads to treatment failure.^[^ ^19^ ^]^ Cell lineage tracking techniques and single‐cell histology have significantly elucidated the clonal heterogeneity within tumors.^[^ ^20^ ^]^ The AVIV team had identified the c‐DTP subset within the DTP population, which is characterized by the ability to proliferate early in drug therapy.^[^ ^6^ ^]^ The DTP cells can survive initial drug treatment through epigenetic adaptation, and escape drug pressure before the emergence of permanent resistance. Both persister cell subsets may serve as the progenitors of drug‐resistant clones that drive tumor recurrence.^[^ ^21^ ^]^

In this study, we established DTP and c‐DTP clones of PC9 cells with first‐generation (gefitinib) or third‐generation (osimertinib) EGFR‐TKIs to identify common metabolic changes and pathways for therapeutic targeting. The cell surface marker DPP4 was significantly upregulated in the DTP and c‐DTP cells, as well as in the tumor tissues from lung cancer patients treated with EGFR‐TKIs. Therefore, targeted inhibition of DPP4 may delay resistance to EGFR‐TKIs and eradicate residual tumor cells.

DPP4, a potential cancer biomarker and therapeutic target,^[^ ^22^ ^]^ is expressed by various primary and metastatic tumors.^[^ ^23^ ^]^ It is a transmembrane enzyme involved in glucose metabolism, cell signaling, differentiation, oxidative stress and the immune system.^[^ ^24^ ^]^ The DPP4 inhibitor sitagliptin is widely used as a hypoglycemic drug,^[^ ^25^ ^]^ and has recently gained interest as a new therapeutic agent for cancer.^[^ ^26^ ^]^ Sitagliptin can also block epithelial cell transformation and breast epithelial cell tumorigenesis by inhibiting PIN1 expression.^[^ ^27^ ^]^ Another study showed that sitagliptin improved the outcomes of immunotherapy in hepatocellular carcinoma patients by up‐regulating CXCL10.^[^ ^28^ ^]^ In addition, monoclonal antibodies targeting DPP4 have been effective against melanoma, renal cell carcinoma, and urothelial carcinoma, while maintaining a favorable safety profile.^[^ ^29^ ^]^

Our findings suggest that DTP and c‐DTP cells undergo metabolic reprogramming, switching from glycolysis to OXPHOS as the main source of energy, to mitigate the stress induced by EGFR‐TKIs. This reprogramming is mediated by the DPP4/MEK/c‐MYC pathway, which upregulates CPT1A and increases fatty acid uptake and FAO within the mitochondria. Recent studies have demonstrated that c‐Myc activation in lung cancer cells upregulates CPT1A and further enhances the antioxidant capacity of cells through the Nrf2 pathway.^[^ ^30^ ^]^ The c‐DTP cells exhibited a more pronounced increase in fatty acid uptake and OXPHOS compared to DTP cells, mostly likely due to activation of the c‐MYC pathway and increased CPT1A expression. The c‐DTP cells also exhibited lower oxidative stress compared to the DTP cells in response to the EGFR‐TKIs due to the up‐regulation of Nrf2, and higher mitochondrial activity. The G1‐phase arrest in DTP cells can be attributed to elevated ROS levels and subsequent activation of the p53 pathway. On the other hand, c‐DTP cells avoid cell cycle arrest due to the high expression of Nrf2, which suppresses p53 activation and allows continued proliferation.

Despite the inherent differences between DTP and c‐DTP cells, DPP4 inhibition effectively down‐regulated downstream pathways, reduced fatty acid uptake and decreased OXPHOS in both subsets. Furthermore, sitagliptin synergized with osimertinib to further diminish the residual persister cells. This synergy was observed across multiple EGFR‐TKIs, including gefitinib, erlotinib, afatinib, almonertinib, lcotinib and cancertinib. While sitagliptin treatment alone exhibited low toxicity in vitro and was not significantly more effective than osimertinib monotherapy in vivo, the combination of both provided superior tumor inhibition compared to osimertinib alone.

The efficacy of sitagliptin should not be measured solely by assessing tumor regression, as residual persister cells represent only a small fraction of the tumor cell population. Clinically, they are closest to the MRD or to the point of maximum tumor shrinkage prior to eventual tumor progression.^[^ ^21^, ^31^ ^]^ Given that sitagliptin may only impact a small subset of cancer cells, its effects take time to manifest. We therefore reassessed its efficacy in MRD and recurrence models, and observed a marked increase in DPP4 expression in the MRD tissues treated with osimertinib alone. In addition, the combination of sitagliptin and osimertinib reduced DPP4 levels and inhibited MRD. Furthermore, compared to osimertinib monotherapy, the combination therapy delayed recurrence and extended survival of the tumor‐bearing mice following drug withdrawal.

## 4 Conclusion

In summary, DPP4 was a significantly up‐regulated marker in both DTP and c‐DTP cells, with elevated levels also observed in patient tumor tissues treated with EGFR‐TKIs. Inhibition of DPP4 blocks high levels of oxidative phosphorylation and high dependence on fatty acid metabolism in persister cells to delayed recurrence. Our study provides novel insights into the mechanisms underlying drug resistance in lung cancer and proposes a promising therapeutic strategy to prevent recurrence.

## 5 Experimental Section

### Cell Culture

EGFR mutated non‐small cell lung cancer cells line (PC9) was cultured in RPMI‐1640 medium supplemented with 10% fetal bovine serum (FBS). The LLC‐2 mouse lung cancer cell line was grown in DMEM supplemented with 10% FBS. Each cell line was maintained at 37 °C in a 5% CO2 atmosphere. The cell lines were identified by STR fingerprinting.

### Persister Cells Derivation and Treatments

Persister cells were using IC90 concentrations from the treatment of EGFR‐mutated non‐small cell lung cancer for 14 days with 300 µM osimertinib or 500 µM gefitinib, respectively.^[^ ^6^ ^]^ Cells were kept under continuous drug exposure during subsequent treatments by replenishing fresh drug to the culture medium every 3 days. Surviving cells were sorted and propagated in drug‐free medium for 20 generations before starting the assay. The number of cells, proliferative capacity, and IC50 after treatment were determined by quantifying the number of cells by full‐field single‐cell imager (Celigo).

### Drug‐Resistant Cell Lines Construction

PC9 cells in logarithmic growth phase (60% – 80% fusion) were supplemented with gefitinib or osimertinib at an initial concentration of 1/10 of the IC50 of the parental cell line for 24 h.^[^ ^32^ ^]^ The medium was discarded, washed twice with PBS, and replaced with drug‐free medium. After the cells resumed growth, the cells were treated at low concentrations for 24 h. After the cells proliferated to normal morphology, the above drug treatments were repeated for 6–8 times at each concentration. When the cells grew stably at this concentration, the culture was continued by increasing the drug concentration 1.2‐1.5 times. Drug induction was continued for 6 months until the cells were able to grow stably at the drug concentration.

### Single‐Cell RNA‐Seq Data Analysis

Single‐cell sequencing raw data were downloaded from GSE243569, including samples YU‐003 and YU‐006.^[^ ^33^ ^]^ To ensure data quality, genes expressed in at least three cells were included. Cells were selected based on the number of feature genes, ranging from more than 200 to fewer than 7000. The single‐cell expression data were normalized using the NormalizeData function from the Seurat package. The linear dimensional reduction was then performed using Principal Component Analysis (PCA), utilizing the first 50 principal components to cluster the cells. The clustering resolution was set to 0.4. The clustering results were visualized using Uniform Manifold Approximation and Projection (UMAP) plots.

### Pathway Analysis

Pathway analysis was used to find out the significant pathway of the marker genes and differentially expressed genes according to MSigDB database. The Fisher's exact test was used to select the significant pathway, and the threshold of significance was defined by p‐value and FDR.

### Go Analysis

Representative samples of DTP and c‐DTP clones were selected for transcriptome sequencing, and a comprehensive method based on Resnik and Lin was adopted for analysis using REVIGO, and then R language was used for mapping. Significantly GO terms in biological process category were enriched in DTP cells and c‐DTP cells. The enrichment value is represented by the color intensity. The p‐value is represented by the circle size.

### CDX Model

2 × 10^6^ PC9 cells were injected subcutaneously into the flanks of 4‐week‐old nude mice (at least 6 per group). When the volume of tumors reached 50–100 mm^3^, mice were orally administered osimertinib or peritumorally injected with sitagliptin 5 times per week. Osimertinib was administered orally at a concentration of 50 mg kg^−1^. Sitagliptin was administered peritumorally injected at a concentration of 30 mg kg^−1^. Tumors were measured with calipers 3 times per week, and tumor volume was calculated as width^2^ × length/2. Mice were executed after 28 days. Tumors were collected and weighed for qRT‐PCR, flow cytometric analysis, and western blot analysis.

### Orthotopic Lung Cancer Model

C57BL/6J mice were inoculated with 1 × 10^6^ LLC/2 cells in the tail vein and given an oral dose of 25 mg kg^−1^ of osimertinib for 5 consecutive days per week (5 days on /2 days off) when the tumor size reached about 100 mm3. Treatment continued for 4 weeks and then the lungs were removed to obtain MRD tissues. The recurrence model was obtained after five weeks of tumor regrowth after treatment withdrawal. The mice were measured by CT at least once before treatment and before sacrifice.

### Analysis of Tumor Recurrence

To track tumors by time and volume measurements, CT scan of the mice chest was used under isoflurane anesthesia. For image processing, RespGate v0.3c was used for breath gating, and NRecon v1.6.10.4 was used for Z‐stack image reconstruction. The combined software CT‐Analyser v1.10.11.0+ and DataViewer v1.5.2.4 were used to view and calculate the image tumor volume. Tumor volume was plotted as a percentage of the volume measured at the start of osimertinib treatment.

### RNA Extraction and Quantitative Real‐Time RT‐PCR (qRT‐PCR)

TRIzol reagent (Invitrogen, Carlsbad, CA) was used to extract total RNA from frozen tumor tissues and cell lines. Using an ABI PRISM Q7 Real‐Time PCR System, quantitative PCR was carried out using SYBR Select Master Mix (Roche, Switzerland) and gene‐specific primers. An SYBR® Prime ScriptTM RT‐PCR kit (Takara Biochemicals, Tokyo, Japan) was used to reverse‐transcribe cDNA from 1 µg of RNA.Using the comparative Ct (the threshold cycle) approach, the quantification of the mRNA was computed as follows: Ratio = 2−∆∆CT = 2−[∆Ct(sample)‐∆Ct(calibrator)], where ∆Ct is the target gene's Ct minus the endogenous control gene's Ct (β‐actin).

### Antibodies and Immunoblotting

Aliquots of 25 µg of protein were separated by SDS polyacrylamide gel electrophoresis (Bio‐Rad, Hercules, CA). Electrophoresed protein samples were transferred to a polyvinylidene difluoride membrane (Bio‐Rad). After three washes, the membranes were incubated for 1 h at room temperature and overnight at 4 °C in blotting grade blocker (Bio‐Rad) with primary antibodies including DPP4, Nrf2, t‐MEK, p‐MEK, t‐ERK1/2, p‐ERK1/2, p53, p21, Cyclin D1, CPT1A, and β‐actin (1:1000 dilution). After rinsing three times, incubate with enzyme‐labelled species‐specific secondary antibodies for 1 h at room temperature. Immunoreactive bands were visualized using SuperSignal West Dura Extended Duration Substrate Enhanced Chemiluminescent Substrate (Pierce Biotechnology). Each experiment was performed independently at least three times.

### Flow Cytometry

For cell surface staining (DPP4), single cell suspensions were incubated for 30 min at four degrees with antibody, from Proteintech. For JC‐1 assay, take appropriate amount of JC‐1 (200 µM) and return it to room temperature, add 10 µL of JC‐1 (200 µM) to each well of medium with a final concentration of 2 µM, shake gently and mix well, and incubate for 15–20 min at 37 °C in a cell incubator. The green fluorescence can be detected by FITC channel, and the red fluorescence can be detected by PE/PI channel. Data were obtained on Fortessa LSRII (BD Biosciences) and analysed using FlowJo.

### Histology

Lung cancer tissues from mice or patient samples were fixed in 10% formalin overnight, then sectioned and stained for H&E and staining immunofluorescence. For H&E staining assay, the tissue sections were degummed in xylene, rehydrated continuously through ethanol and rinsed in PBS. Antigens were microwaved in a special antigen recovery solution at 95 degrees Celsius for 15 min and then naturally cooled to room temperature. To block non‐specific signals, 5% goat serum was used and added to PBS. For immunofluorescence staining (IF) assay, slides were stained with the indicated primary antibodies. After overnight incubation, slides were washed and stained with secondary antibodies for 60 min at room temperature. Samples were mounted on microscope slides using an extended Antifade with DAPI and imaged using an Olympus A1Si laser scanning confocal microscope. For immunohistochemical staining (IHC) assay, slides were washed with PBS and quenched with buffer containing 10% methanol quenched with 0.1% H_2_O_2_ for 30 min. After blocking with 5% goat serum for 1 h at room temperature, target‐specific antibodies were added and incubated overnight at 4 °C, followed by reaction with biotinylated secondary antibodies to capture IHC and multiple IHC signals using a Leica Aperio Versa 8.

### Energy Metabolism Detected by Seahorse

To determine the glycolysis rate, the Seahorse XF glycolysis rate assay uses ECAR and OCR measurements to determine the cells glycolysis proton outflow rate (glycoPER). First, parental, DTP, c‐DTP, and drug‐resistant cells were incubated in Seahorse XF glycolytic rate assay solution containing glucose, glutamine, sodium pyruvate, and HEPES buffer, recording the base rate of the three measurement cycles. Subsequently, Rot/AA (mitochondrial electron transport chain inhibitors) is added to inhibit mitochondrial oxygen consumption (and therefore CO2‐derived protons). The second dose adds 2‐deoxyd‐glucose (2‐DG), a glucose analogue that inhibits glycolysis by competitively binding to the first enzyme in the glycolytic pathway, glucohexokinase. The resulting reduction in PER provides qualitative confirmation that the PER produced before the addition of 2‐DG is primarily due to glycolysis.​

To assess changes in oxygen consumption rate (OCR) and cell substrate oxidation dependence in living cells, Seahorse XF substrate oxidative stress test kit was used to analyze three major mitochondrial substrates: long‐chain fatty acids (LCFAs), glucose/pyruvate (G/P), and glutamine (Q). Parental, DTP, c‐DTP, and drug‐resistant cells were incubated overnight and then subjected to cell mitochondrial stress tests (MST) after adding inhibitors, respectively. Etomoxir inhibits the oxidation of LCFAs by inhibiting carnitine palmitoacyltransferase 1a (CPT1a). UK5099 inhibits oxygen 2 of glucose and/or pyruvate by inhibiting mitochondrial pyruvate carrier protein (MPC); BPTES inhibits the oxidation of glutamine by inhibiting glutaminase 1 (GLS‐1). MST is a recognized powerful tool for studying mitochondrial function, and when used in combination with these inhibitors, it can reveal dependence on specific metabolic substrates.

### Glycolysis/OXPHOS Assay

The Glycolysis/OXPHOS assay kit was purchased from Dojindo. For lactate assay, 20 µL supernatant samples of parental, DTP, c‐DTP, and drug‐resistant cells were added to each well. Each well was added with 80 µL lactate working solution and cultured at 37 °C for 30 min. The absorbance at 450 nm was detected by enzyme‐labeled instrument.

For ATP assay, the parental, DTP, c‐DTP, or drug‐resistant cells suspension (100 µL per well) was added to a white 96‐well plate and 100 µL ATP working solution was added to each well. The orifice plate with ATP working solution added was placed in an enzymoleter set at 25 °C and stood for 10 min. Luminescence (RLU) was detected finally.

### Oxygen Consumption Rate(OCR) Plate Assay

Oxygen Consumption Rate(OCR) Plate Assay Kit was purchased from Dojindo. Parental, DTP, c‐DTP, and drug‐resistant cells were pre‐cultured overnight in a 37 °C and 5% CO2 incubator. The orifice plates were placed into a fluorescent enzyme labeled instrument preset at 37 °C and cultured for 30 min. Mineral oil was dropped into each hole to isolate the air. The continuous reading mode of the enzyme labeler was used for detection every 10 min for 200 min. (Ex: 500 nm, Em: 650 nm, bottom reading mode). The OCR value was calculated by fluorescence intensity.

### Fatty Acid Uptake Assay

Fatty Acid Uptake Assay Kit was purchased from Dojindo. Parental, DTP, c‐DTP or drug‐resistant cells were seeded into well plates or dishes. The medium was removed and washed twice with serum‐free medium. Serum‐free medium was added and cultured at 37 °C and 5% CO2 for 15 min. When the supernatant was removed, the Fatty Acid Uptake Probe working solution was added, and cultured at 37 °C and 5% CO2 for 15 min. After the supernatant was removed, the cells were cleaned with washing buffer solution for 3 times, and the washing buffer solution was added for detection by fluorescence microscope or Celigo.

### Ethics Statement

All clinical studies were approved by the ethics committees of the Shanghai Cancer Institute. Patient samples were obtained from Shanghai Chest Hospital. All animal experimental procedures acquired official approval of the Institutional Animal Care and Use Committee of Model Animal Research Center of Shanghai Cancer Institute and the Institutional Animal Care and Use Committee of Shanghai Institute of Materia Medica, Chinese Academy of Sciences.

## Conflict of Interest

The authors declare no conflict of interest.

## Author Contributions

Y.Z., X.Z., X.Y., and X.C. contributed equally to this work. L.X.Y. designed the study, interpreted the data, and revised the paper. Z.Y.Z. designed, performed experiments, interpreted experimental results, and wrote papers. Bioinformatics data processing was performed by Z.X.J., including RNA‐seq and single cell data analysis. Y.X.P. designed animal experiments and performed data analysis. C.X.S. collected clinical samples and diagnosed µCT results of mice. W.Y.H. assisted with the Seahorse assay and related data analysis. H.J.Y. performed cell culture, siRNA transfection, and rt‐qPCR assay. L.R. directed the administration protocol of mice and edited the paper.

## Supporting information



Supporting Information

## Data Availability

Research data are not shared.
