# Supplementary material for: EGFR‐TKIs Induced DPP4 Drives Metabolic Reprogramming of Persister Cells in Lung Cancer
Source: Adv Sci (Weinh). 2025 Jun 6;12(31):e06950. doi: 10.1002/advs.202506950 (PMC12376602; doi:10.1002/advs.202506950)
Supplement: Supplementary file 1 — Supporting Information [file ADVS-12-e06950-s001.pdf]

ADVANCED SCIENCE
Open Access

# Supporting Information

for *Adv. Sci.*, DOI 10.1002/advs.202506950

EGFR-TKIs Induced DPP4 Drives Metabolic Reprogramming of Persister Cells in Lung Cancer

*Yuanzhou Zhang, Xiaojun Zhang, Xupeng Yang, Xingshi Chen, Yuehong Wang, Jingying Hu, Rui Liu* and Xiaoying Luo**

# SUPPLEMENTARY MATERIALS

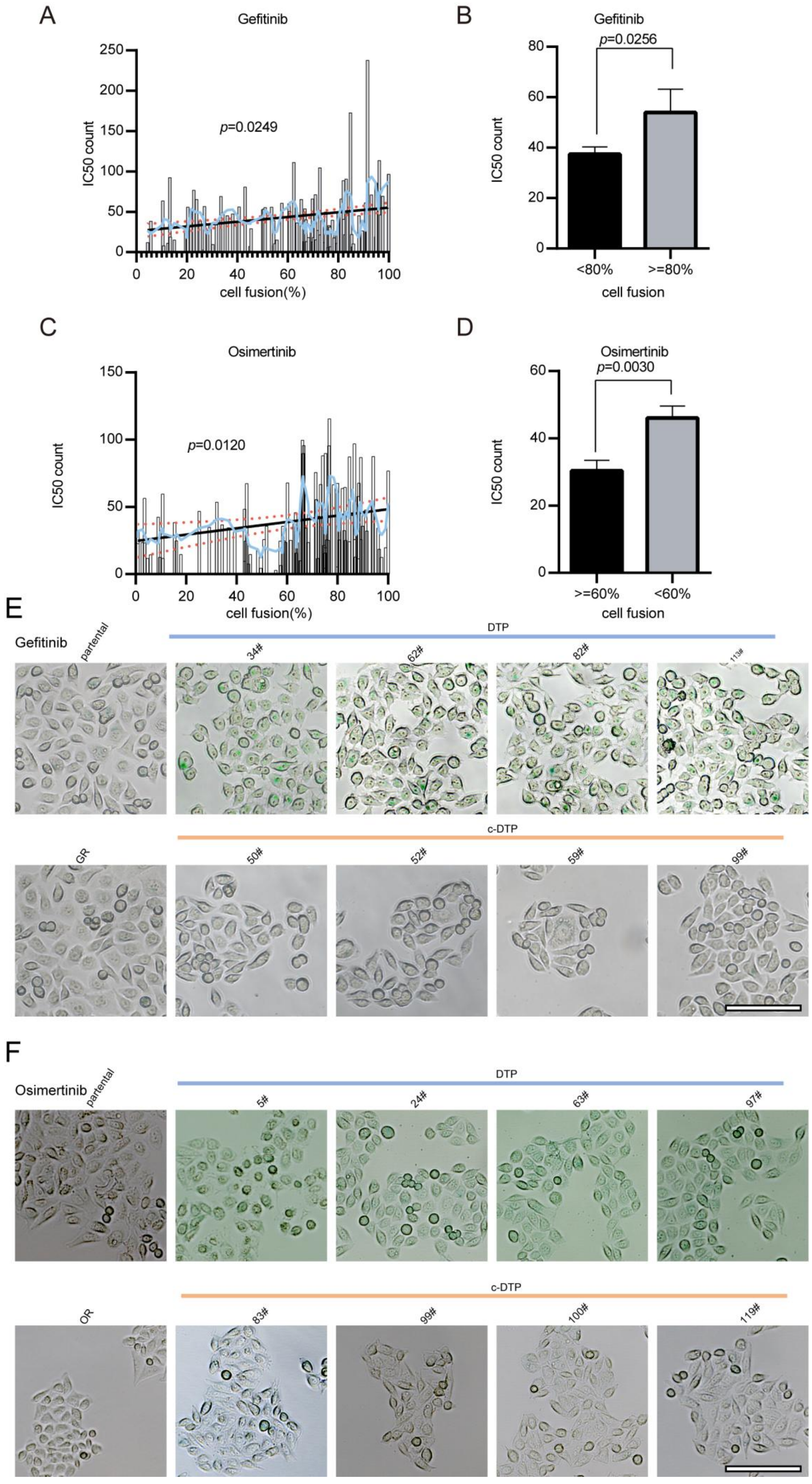


# Figure S1

Persister cells fractionation analysis and identification by β-galactosidase staining. A-D) Correlation analysis of IC50 value and cell fusion degree of persistent cells after gefitinib or osimertinib treatment. Paired Pearson product-moment correlation coefficient test. E and F) Representative β-galactosidase staining results after treatment with gefitinib or osimertinib in parental cells, DTP cells, c-DTP cells and osimertinib /gefitinib-resistant cells. Green represents positive results. Scale bars, 100 μM.

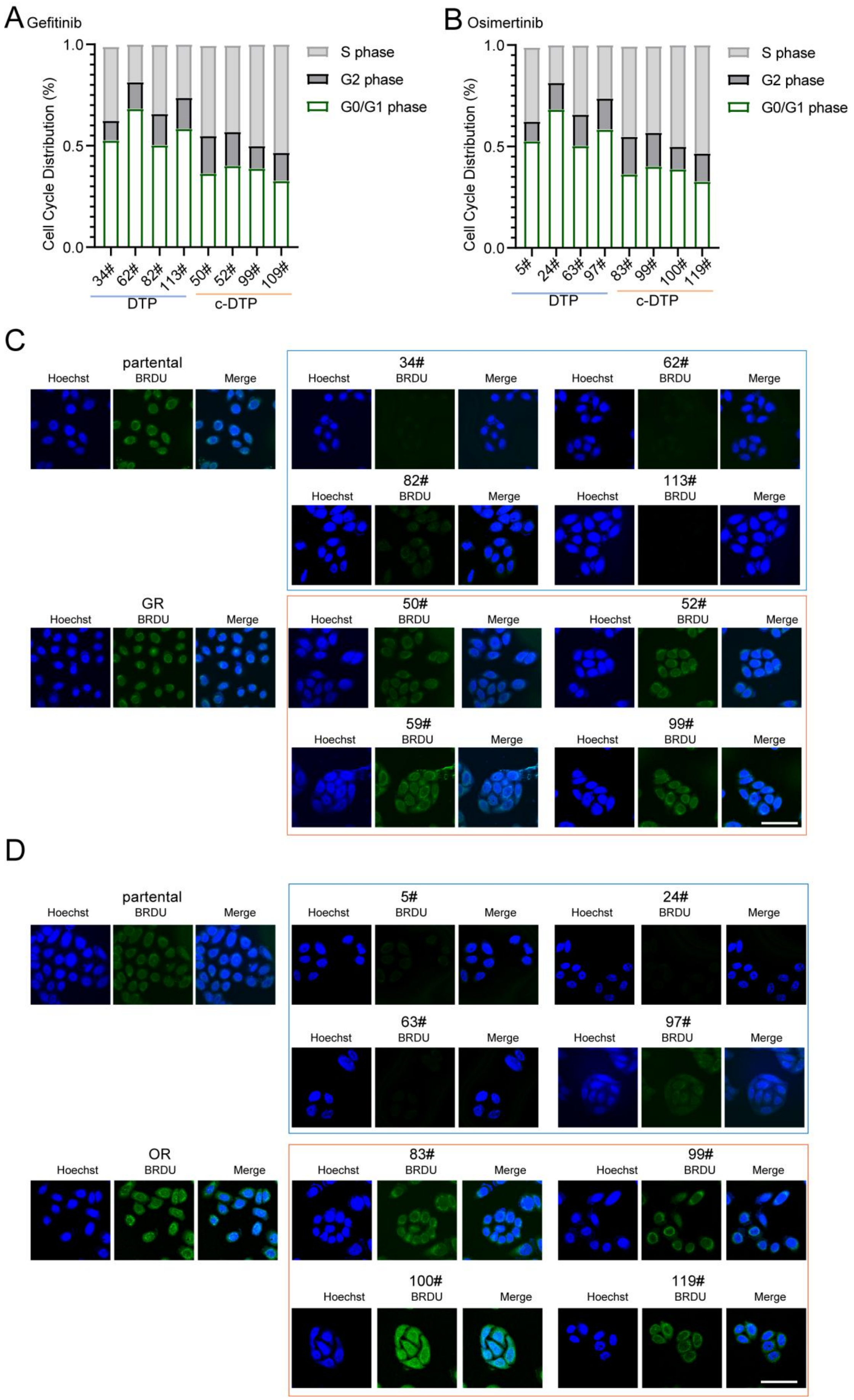

A Gefitinib
Cell Cycle Distribution (%)
1.0
0.5
0.0
34# 62# 82# 113# 50# 52# 99# 109#
DTP
c-DTP
S phase
G2 phase
G0/G1 phase
B Osimertinib
Cell Cycle Distribution (%)
1.0
0.5
0.0
5# 24# 63# 97# 83# 99# 100# 119#
DTP
c-DTP
S phase
G2 phase
G0/G1 phase
C
partental
Hoechst BRDU Merge
34#
62#
82#
113#
GR
50#
52#
59#
99#
D
partental
5#
24#
63#
97#
OR
83#
99#
100#
119#

**Figure S2**

Cell cycle analysis of parental cells, DTP cells, c-DTP cells and osimertinib /gefitinib-resistant cells. A and B) Distribution and quantification of DTP cells and c-DTP cells in cell cycle phases according to expression of cell cycle gene signatures after gefitinib or osimertinib treatment. C and D) Representative image of BRDU fluorescence staining results of parental cells, DTP cells, c-DTP cells and osimertinib /gefitinib-resistant cells after treatment with gefitinib or osimertinib. Scale bars, 50 μM.

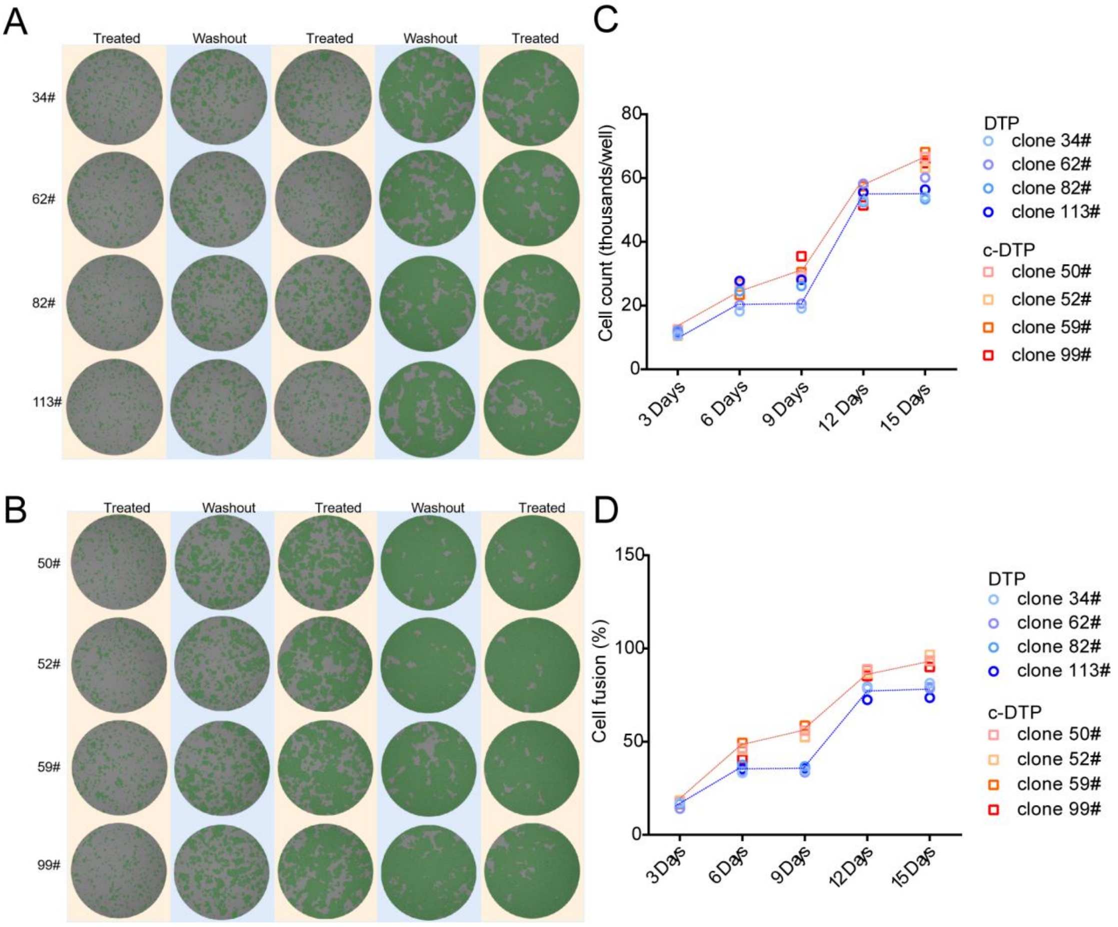


**Figure S3**

Reversibility of cell proliferation in persister cells. A-D) The proliferation capacity of DTP cells was detected in the cell drug treatment stage and the drug removal stage respectively by Celigo Image Cytometer after gefitinib or osimertinib treatment.

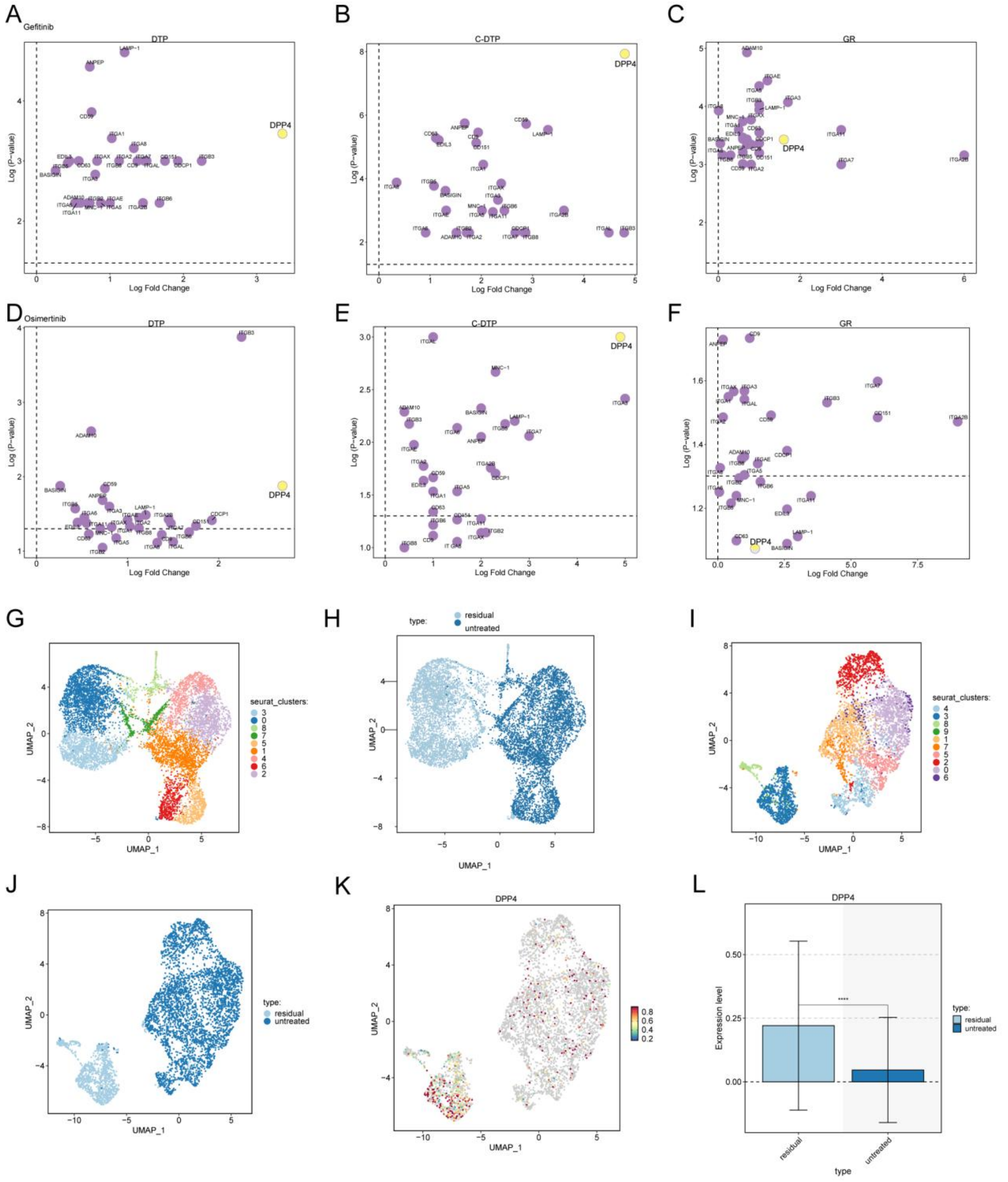

**Figure S4**

Cell surface markers were analyzed with persistent cells and patient

single cell data. A-F) The mRNA expression of 36 cell surface markers in parental cells, DTP cells, c-DTP cells and osimertinib /gefitinib -resistant cell subsets was quantitatively determined by RT-qPCR. Fold change values are calculated relative to parental cells. G) UMAP Visualization of Cellular Subpopulations. H) UMAP plot showing clustering of YU-003 untreated and residual tumor cells by single-cell RNA-sequencing. I) UMAP Visualization of Cellular Subpopulations. J) UMAP plot showing clustering of YU-006 untreated and residual tumor cells by single-cell RNA-sequencing. K) Feature plot depicting expression of DPP4 in individual cells. L) Box plot showing the expression levels of DPP4 in Residual and Untreated groups, with differences tested using the Wilcoxon test.

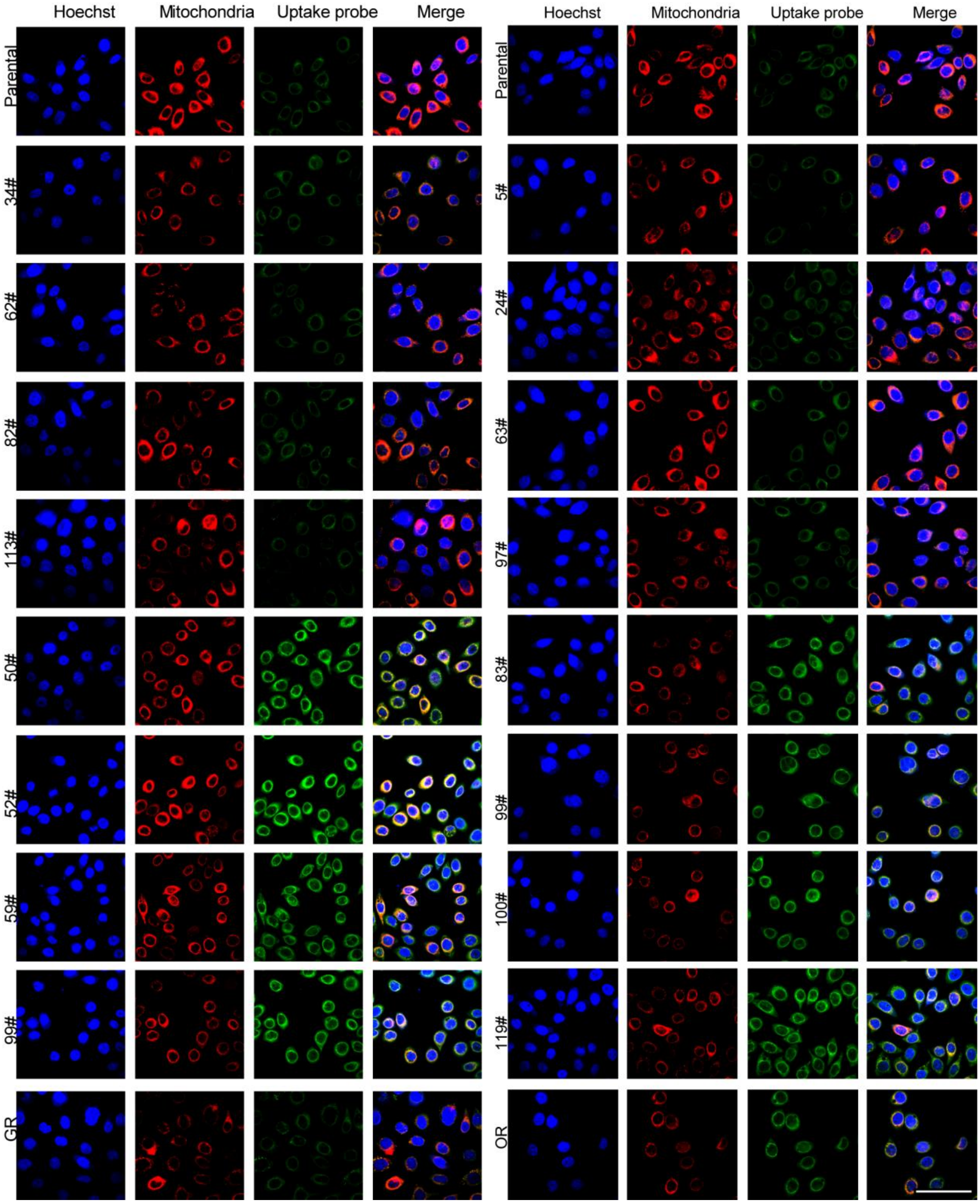


**Figure S5**

Fatty acid uptake analysis of parental cells, DTP cells, c-DTP cells and osimertinib /gefitinib-resistant cells. Representative fluorescence results of parental cells, DTP cells, c-DTP cells and osimertinib /gefitinib-resistant cells were identified with mitochondrial staining (red) and fatty acid uptake probes (green), Scale bars, 100 μM.

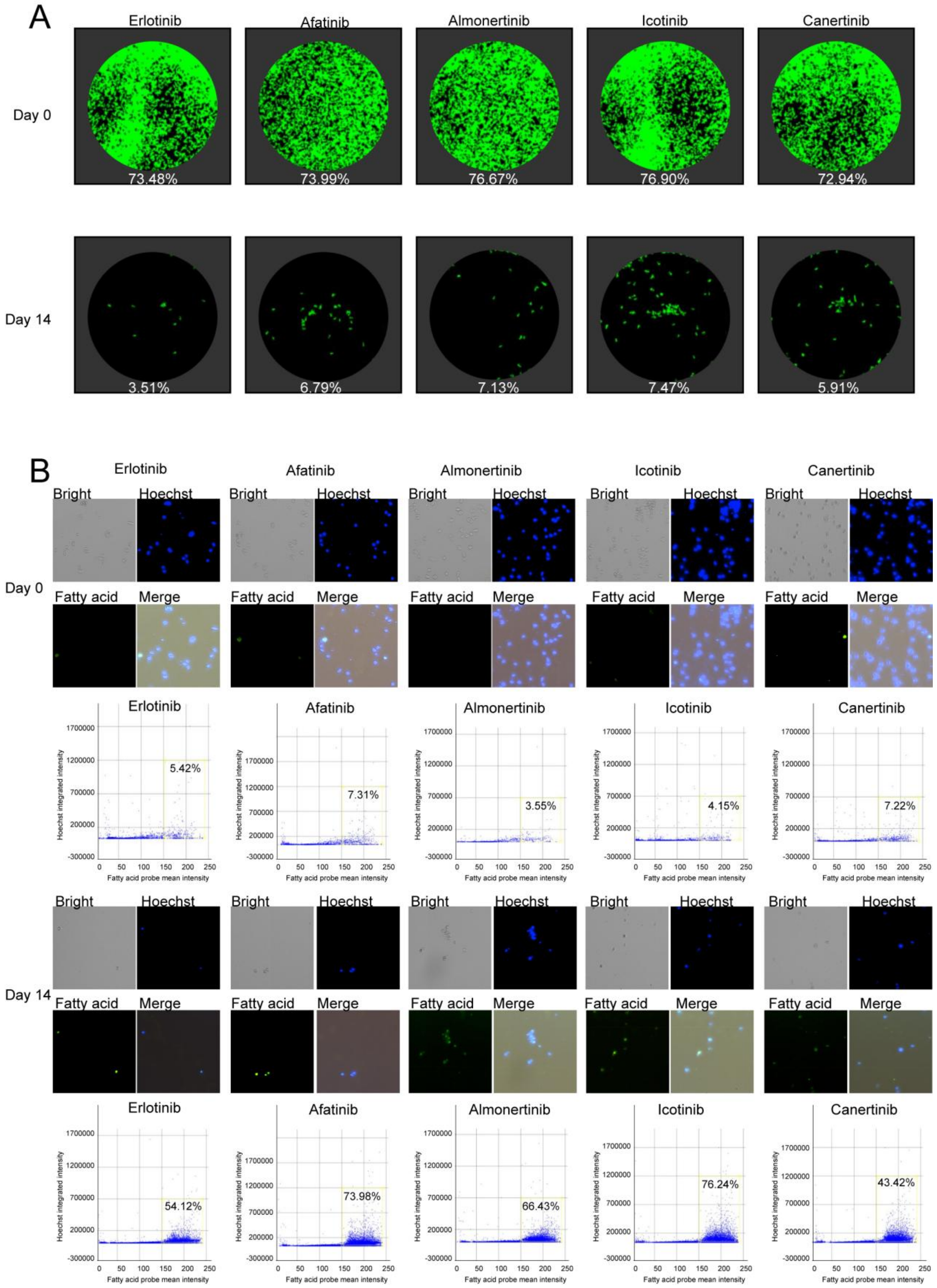


# Figure S6

Fatty acid uptake in persistent cells after multiple EGFR-TKIs treatment were detected. A and B) Fatty acid uptake was detected by fluorescent

probe after treatment with different EGFR-TKIs. Fluorescence results and single cell fluorescence intensity analysis were performed with Celigo Image Cytometer.

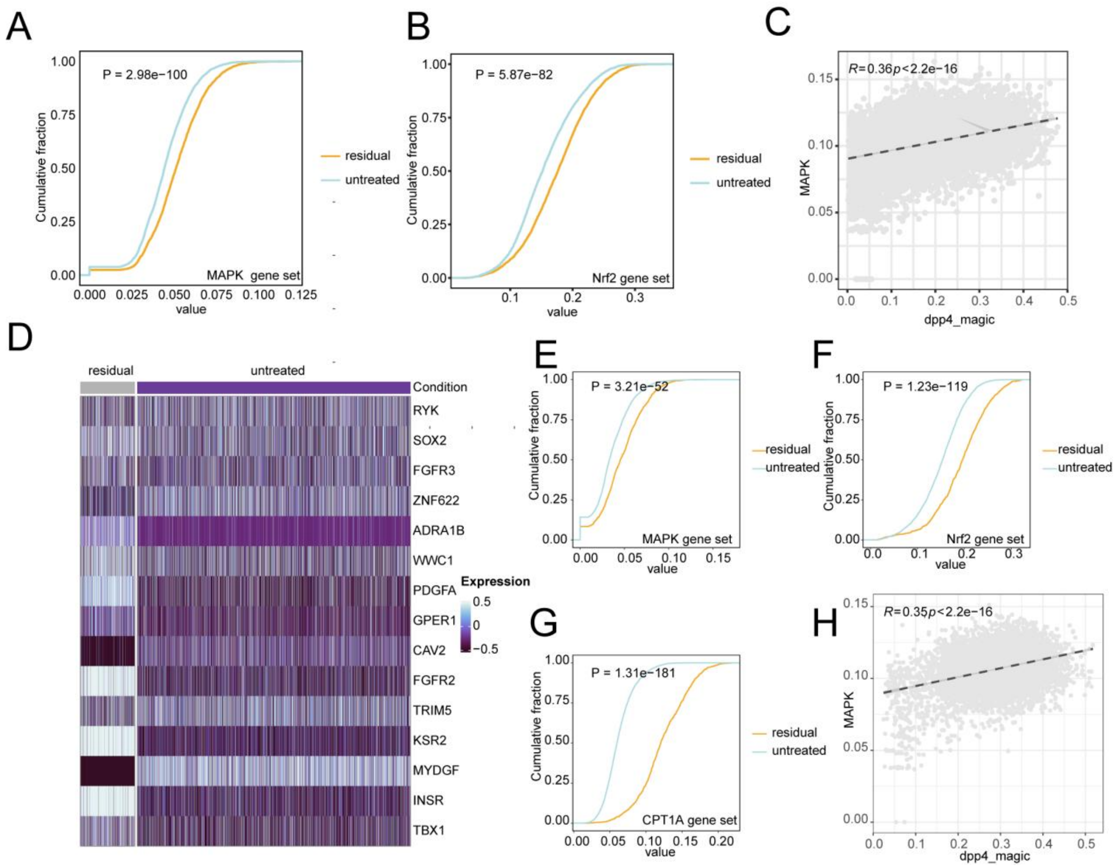


**Figure S7**

Functional enrichment analysis of single cell data sets. A) ECDF (Empirical Cumulative Distribution Function) plot of residual cells from sample_003 compared with untreated cells in MAPK gene set. B) ECDF plot of residual cells from sample_003 compared with untreated cells in Nrf2 gene set. C) Spearman correlation analysis between expression of DPP4 and MAPK pathway in residual cells from sample_003. D) Single cell heat map of MAPK gene set in sample_006. E) ECDF plot of residual cells from sample_006 compared with untreated cells in MAPK

gene set. F) ECDF plot of residual cells from sample_006 compared with untreated cells in Nrf2 gene set. G) ECDF plot of residual cells from sample_006 compared with untreated cells in CPT1A expression. H) Spearman correlation analysis between expression of DPP4 and MAPK pathway in residual cells from sample_006.

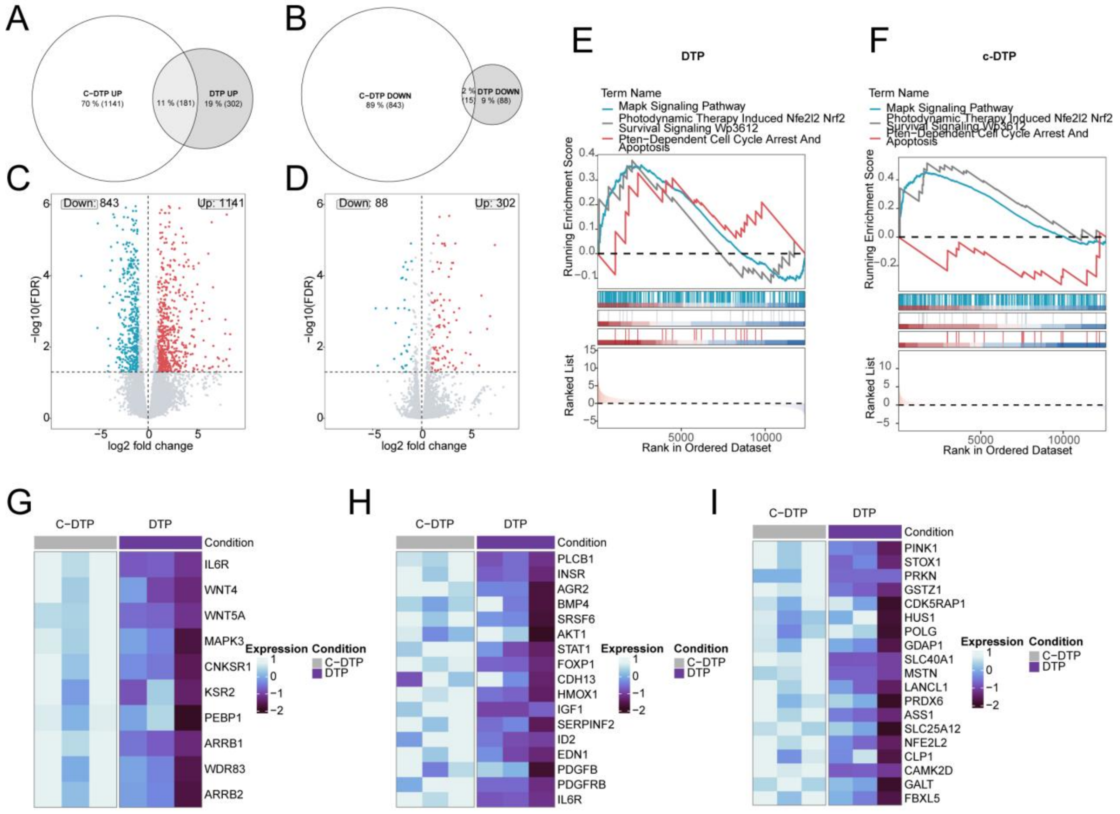


**Figure S8**

Functional enrichment analysis of transcriptome sequencing results from DTP and c-DTP cells. A) Venn diagram of intersection of up-regulated genes in DTP cells and c-DTP cells. B) Venn diagram of intersection of down-regulated genes in DTP cells and c-DTP cells. C) Volcanic map of

differential genes in c-DTP cells. D) Volcanic map of differential genes in DTP cells. E) GSEA enrichment analysis of c-DTP cells. F) GSEA enrichment analysis of DTP cells. G) Heat map of MAPK gene sets in c-DTP cells and DTP cells. H) Heat map of cell proliferation gene sets in c-DTP cells and DTP cells. I) Heat map of oxidative stress gene sets in c-DTP cells and DTP cells.

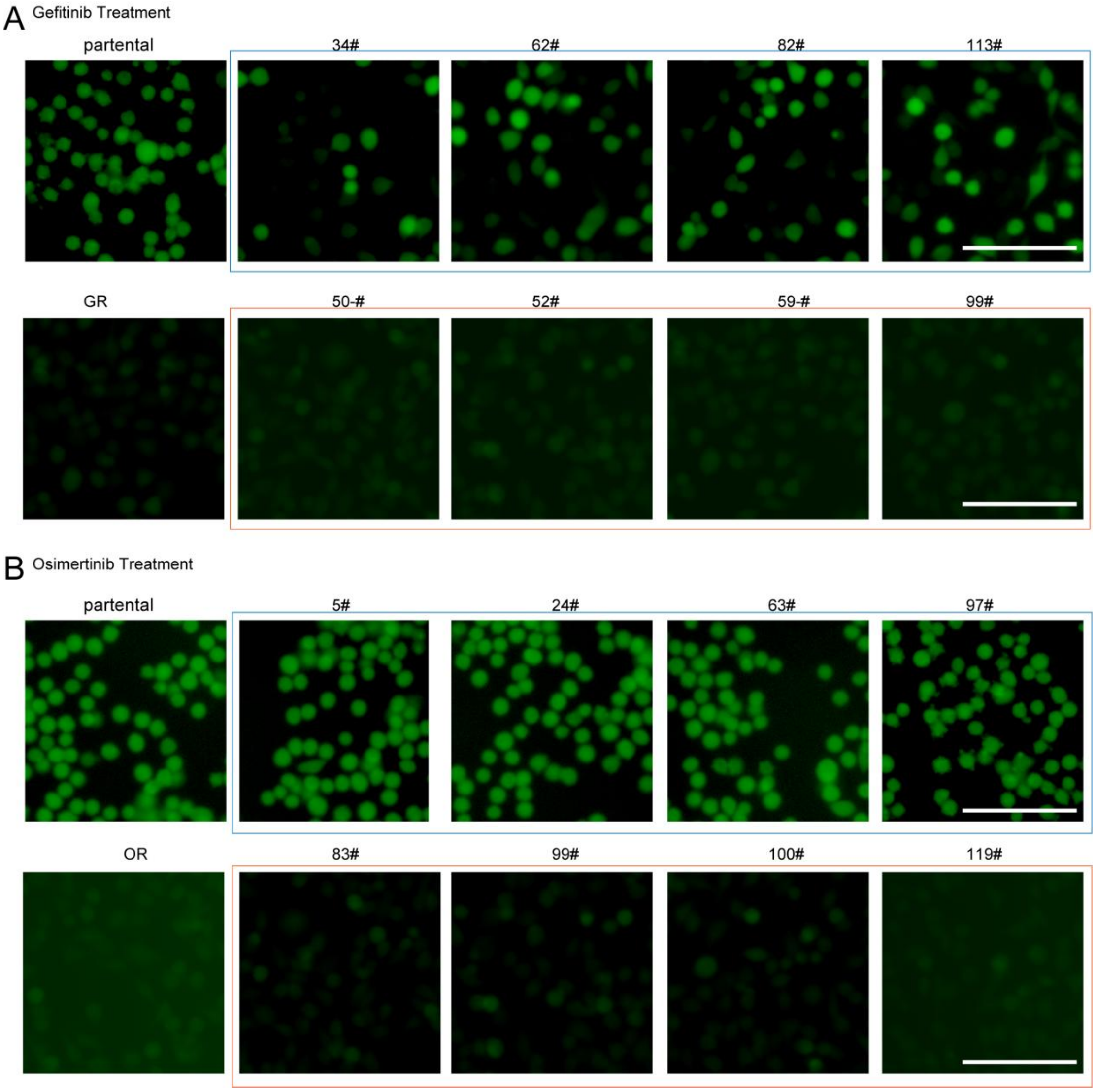


**Figure S9**

ROS fluorescence of parental cells, DTP cells, c-DTP cells and osimertinib /gefitinib-resistant cells. A and B) Representative image of

ROS fluorescence probe intensity detected by fluorescence microscopy, Scale bars, 100 μM.

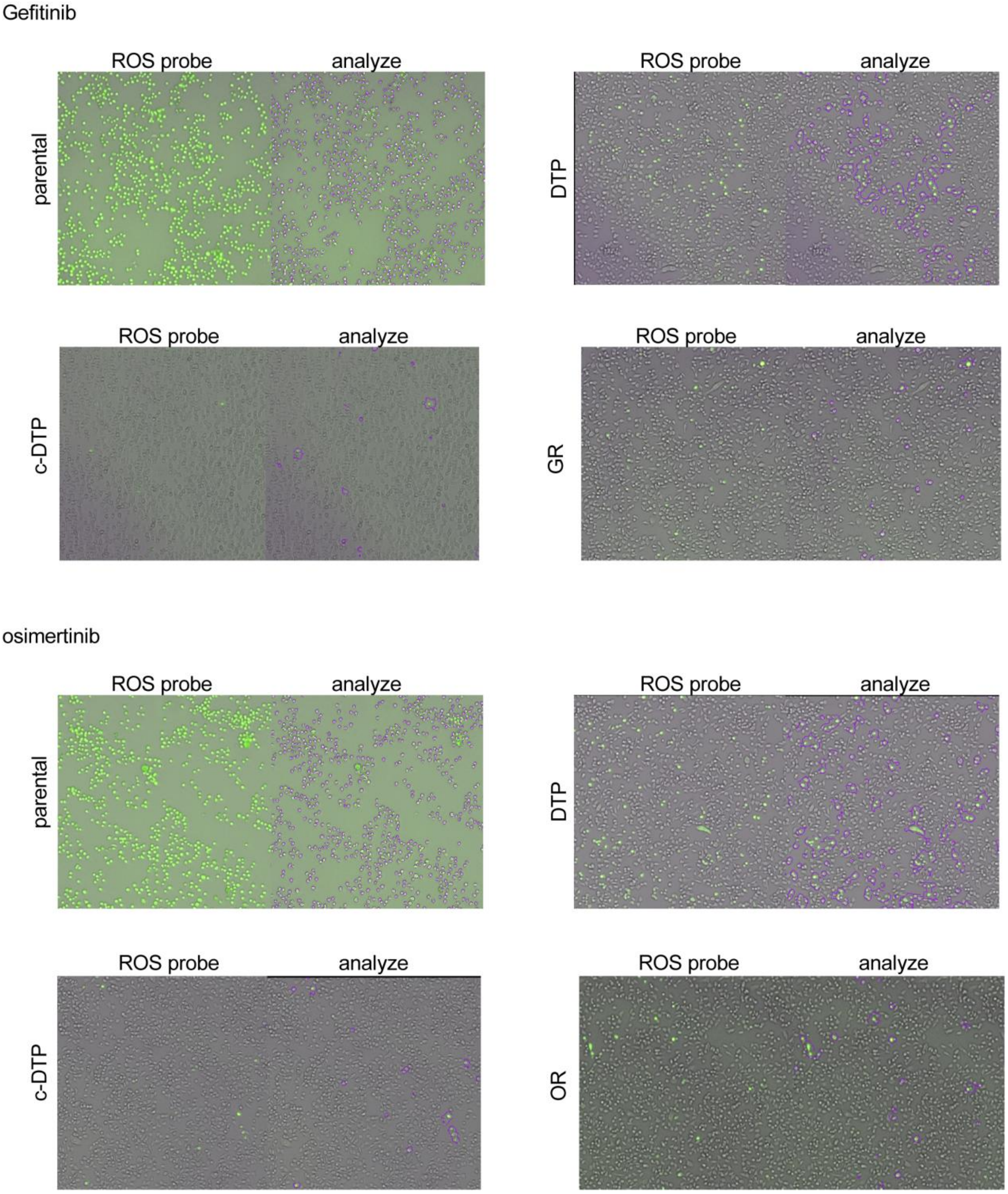


**Figure S10**

ROS fluorescence analysis of parental cells, DTP cells, c-DTP cells and osimertinib /gefitinib-resistant cells. Fluorescence intensity calculation and positive rate of ROS fluorescence probe were detected by Celigo.

Data are representative of three independent experiments.

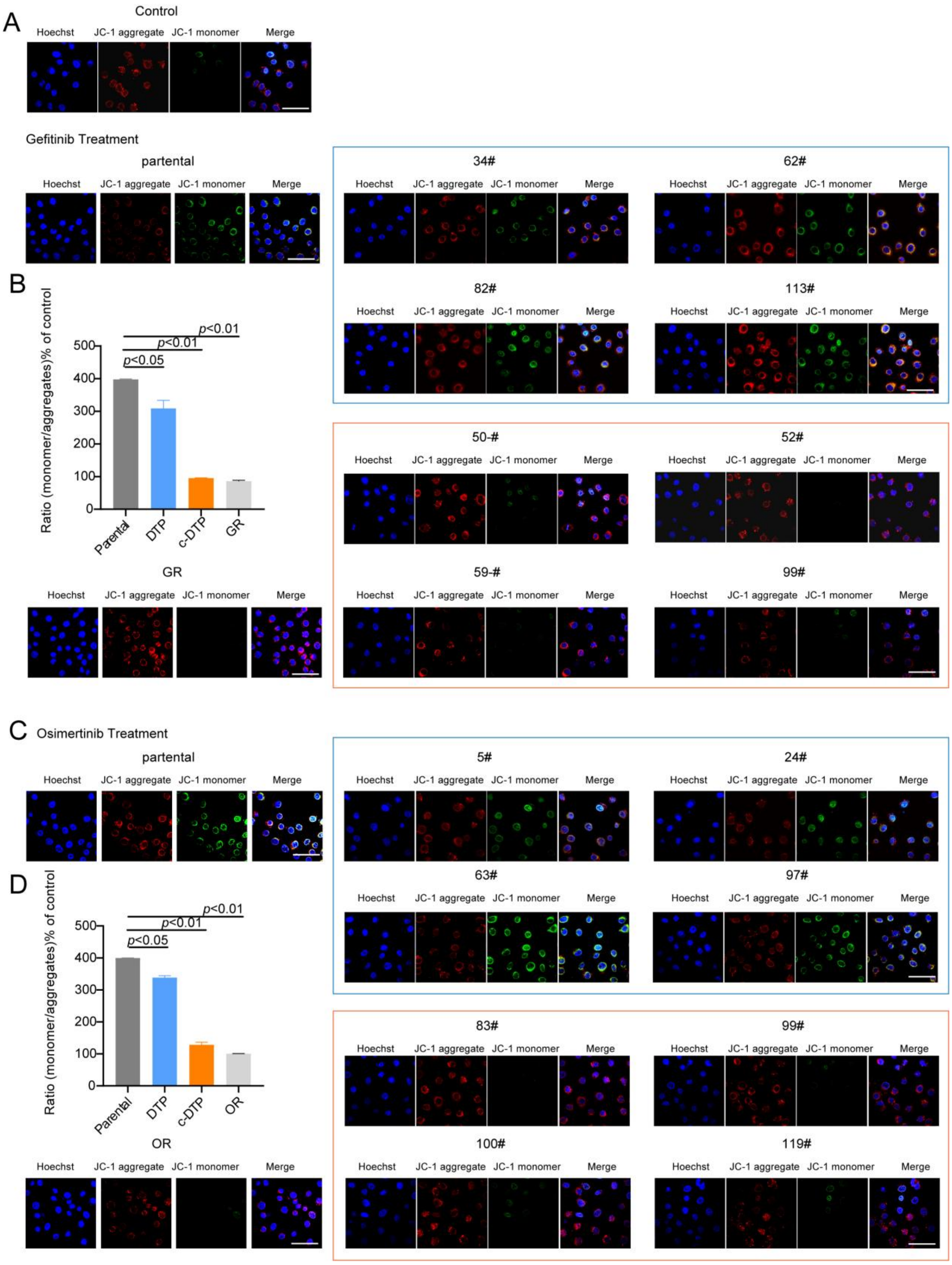


**Figure S11**

Mitochondrial membrane potential was detected by JC-1 staining in

persister cells. A-D) Representative images and analysis of JC-1 aggregate (red) and monomer (green) fluorescence detected by fluorescence microscopy in parental cells, DTP cells, c-DTP cells and osimertinib /gefitinib-resistant

cells.

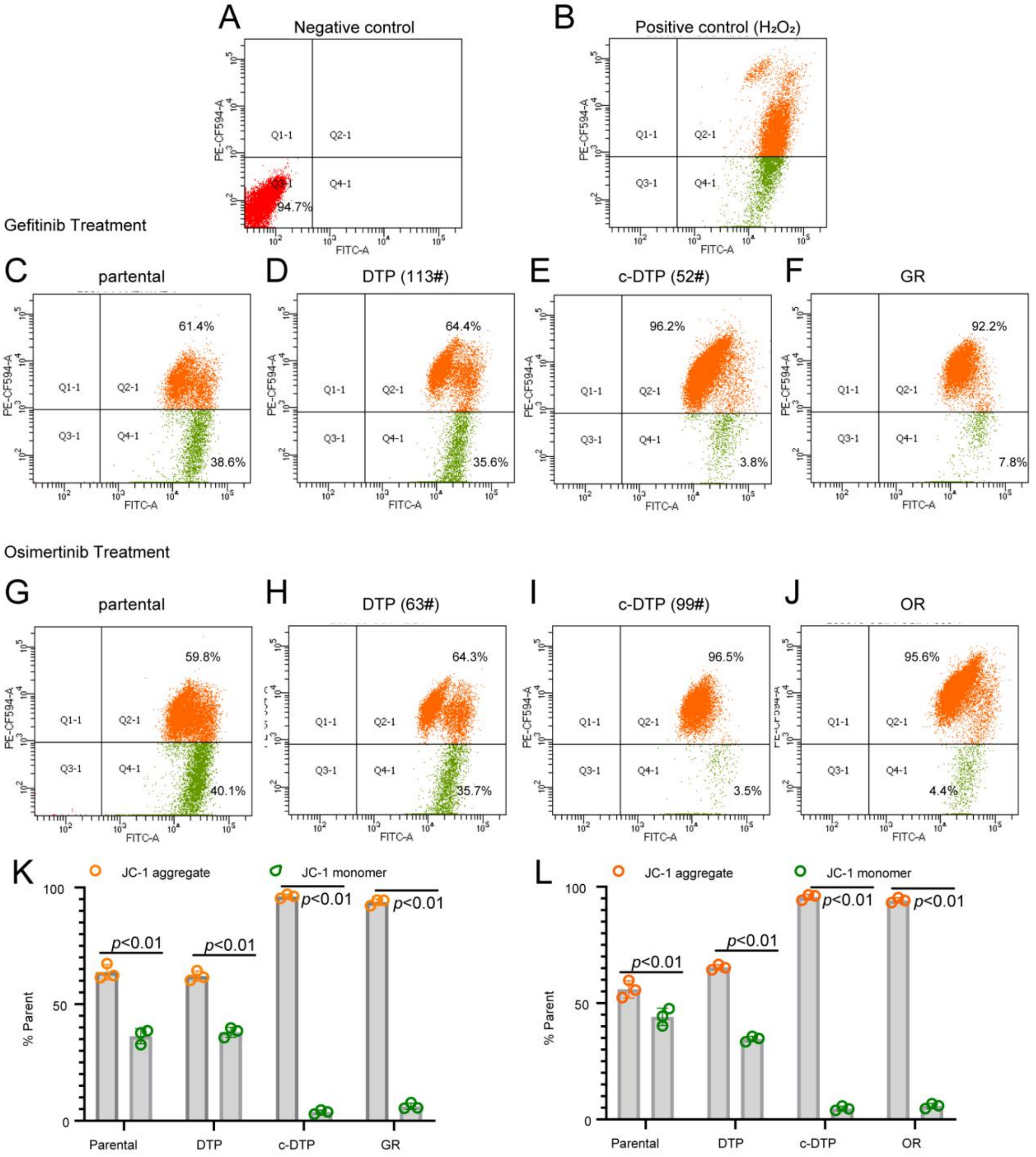


**Figure S12**

Mitochondrial membrane potential were detected by flow cytometry after

JC-1 staining in persister cells. A-L) Distribution and quantification of PC9 cells in Mitochondrial membrane potential according to JC-1 staining after gefitinib or osimertinib treatment.

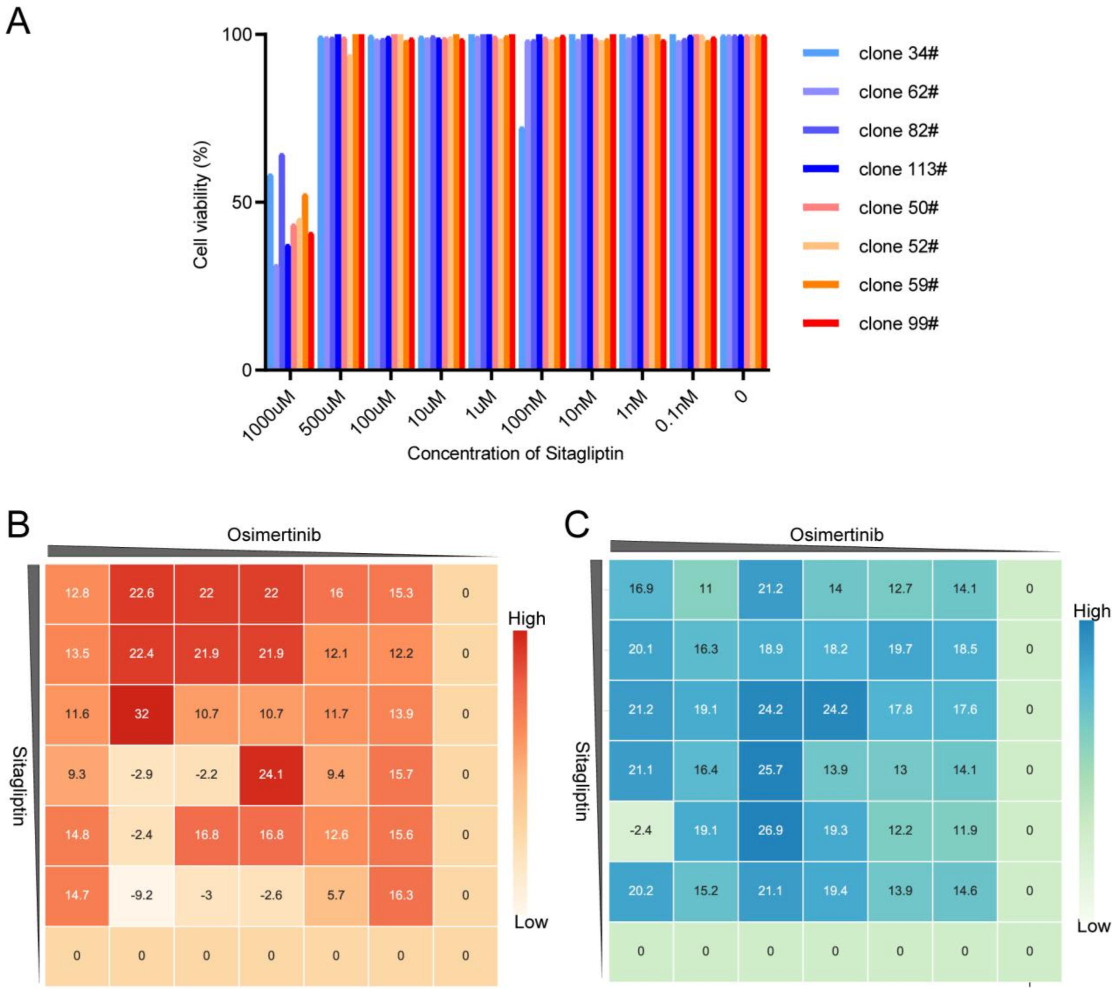


**Figure S13**

Optimal therapeutic concentration of sitagliptin. A) The cell viability of PC9 cells at different sitagliptin treatment concentrations were measured by CCK8. B and C) Heatmaps of bliss synergy scores demonstrated synergistic activities of sitagliptin and EGFR-TKIS in PC9 cells (sitagliptin: 0 , 6.25, 12.5, 25, 50, 100, 200 nM; gefitinib: 0, 6.25, 12.5,

25, 50, 100, 200μM; osimertinib: 0, 6.25, 12.5, 25, 50, 100, 200μM). D) IC50 of gefitinib in parental cells, DTP cells, c-DTP cells and gefitinib-resistant cells was calculated after 72 h of treatment. Data is representative of 2 independent experiments. E) IC50 of osimertinib in parental cells, DTP cells, c-DTP cells and osimertinib-resistant cells was calculated after 72 h of treatment. Data is representative of 2 independent experiments.

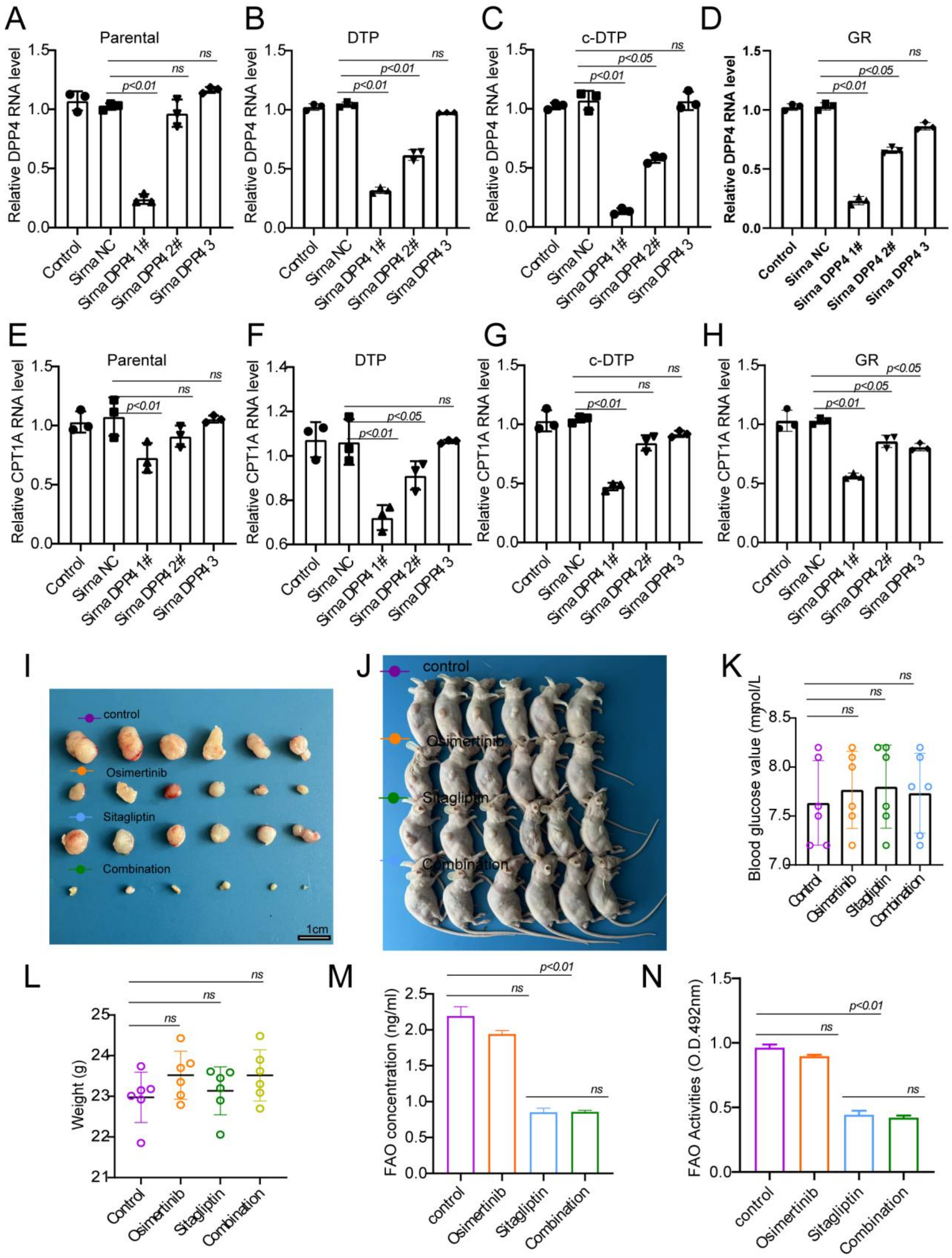


**Figure S14**

Detection of therapeutic effect by DPP4 SiRNA on persistent cells and supplementary data of therapeutic effect of sitagliptin in nude mice. A-H)

The mRNA expression of DPP4 and CPT1A in parental cells, DTP cells, c-DTP cells and gefitinib-resistant cell subsets was quantitatively determined by RT-qPCR. Fold change values are calculated relative to parental cells; *n*= 3, Paired two-tailed Student's t-test. I) Subcutaneous PC9 tumors treated with osimertinib, sitagliptin, the combination or control. Scale bars, 1 cm. J) PC9 subcutaneous xenograft growth in nude mice; n= 6 mice. K) Blood glucose value of nude mice; n= 6 mice. L) Weight of nude mice treated with osimertinib, sitagliptin, the combination or control. M) According to the FAO assay kit, the substrate of tissue homogenisation was detected by enzyme-labeled assay, *n*= 3, Paired two-tailed Student's t-test. Data represented as mean ± SEM. N) According to the Fatty acid oxidation assay kit, the substrate of tissue homogenisation was detected by enzyme-labeled assay, *n*= 3, Paired two-tailed Student's t-test. Data represented as mean ± SEM.

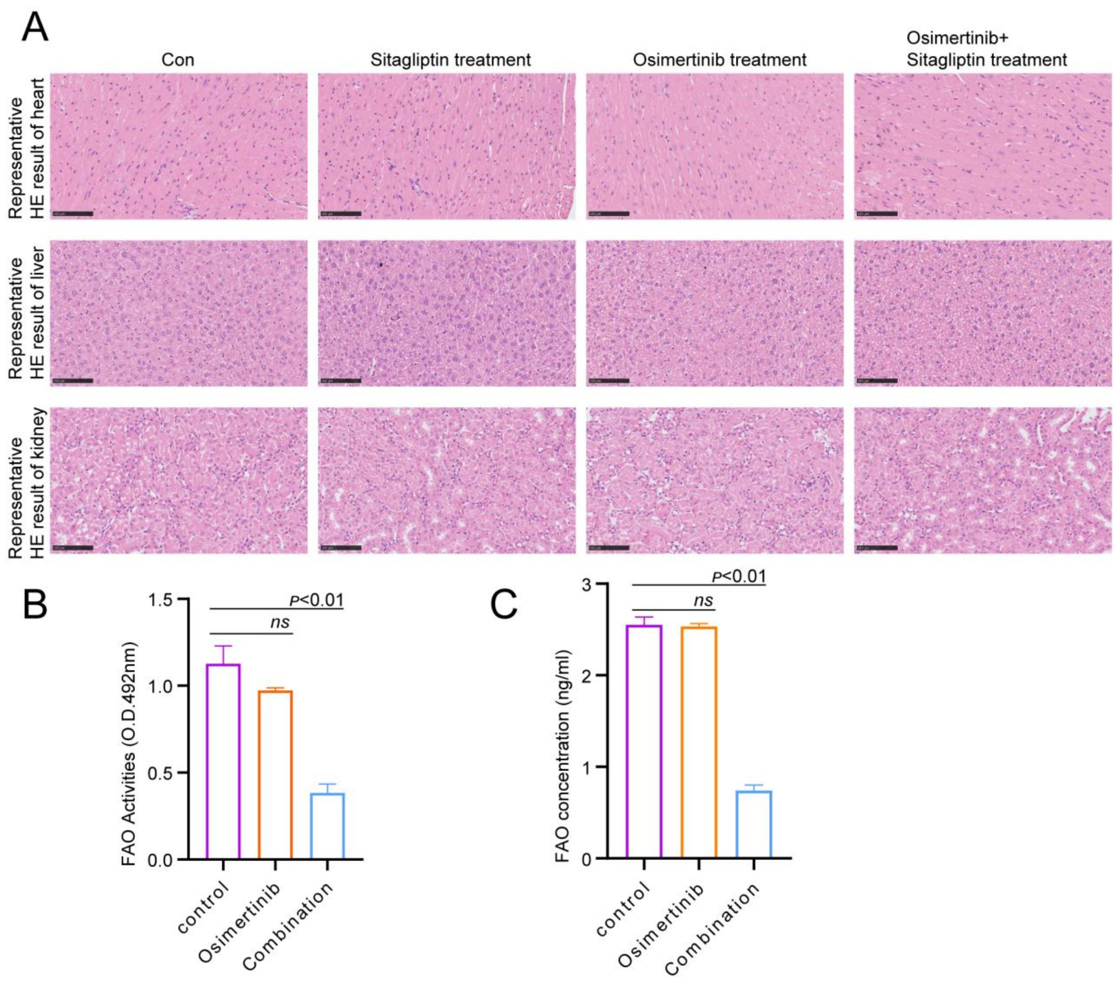


**Figure S15**

Pathological examination of major organs after treatment and detection of fatty acid oxidation in tumor tissues. A) Representative HE results of heart, liver and kidney in nude mice treated with osimertinib, sitagliptin, the combination or control, Scale bars, 100 μM. B) According to the Fatty acid oxidation assay kit, the substrate of homogenisation in tumor tissues was detected by enzyme-labeled assay, *n*= 3, Paired two-tailed Student's t-test. Data represented as mean ± SEM. C) According to the FAO assay kit, the substrate of homogenisation in tumor tissues was detected by

enzyme-labeled assay, *n*= 3, Paired two-tailed Student's t-test. Data represented as mean ± SEM.

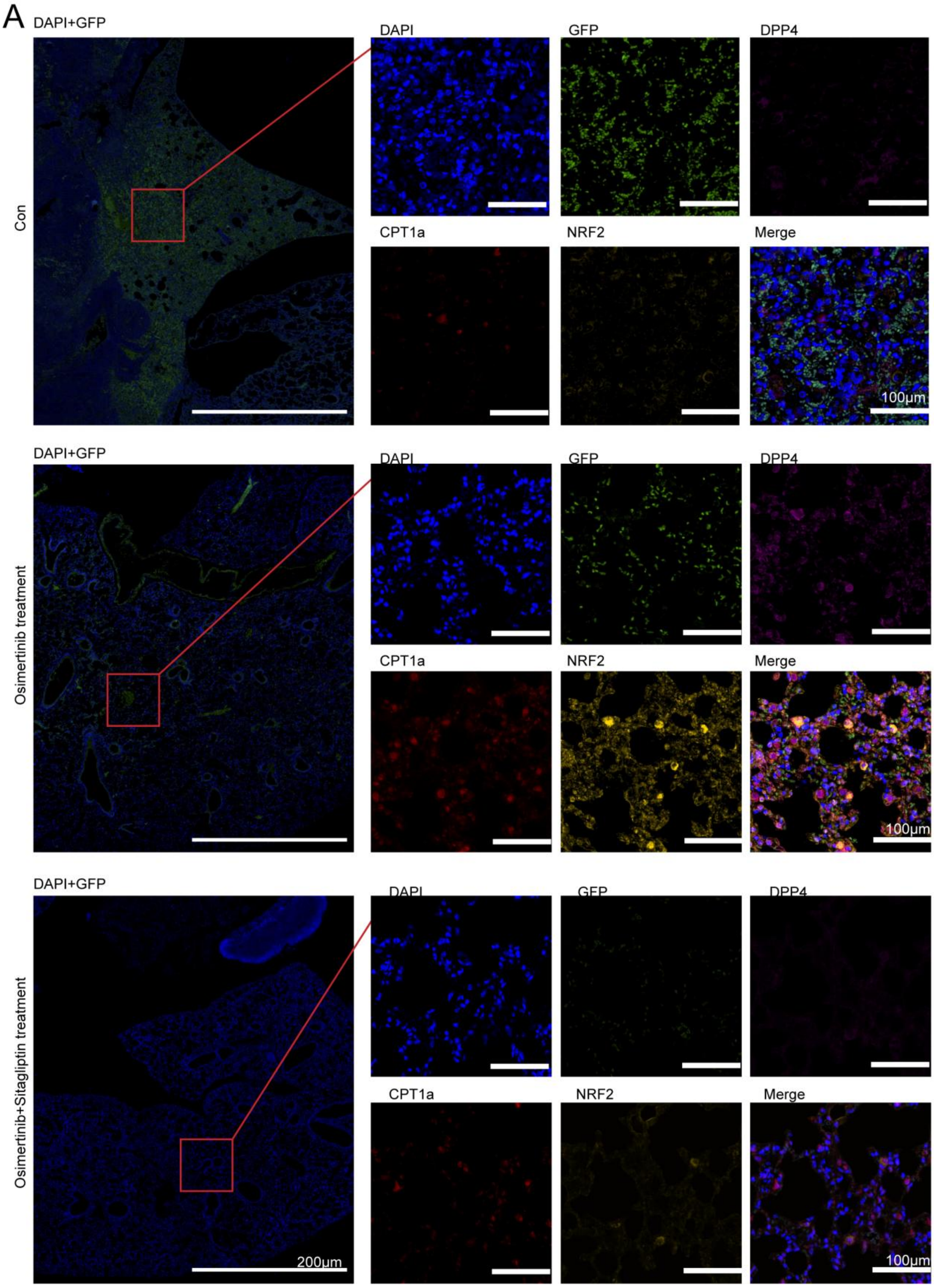


**Figure S16**

Specific expression of DPP4, NRF2 and CPT1A in umour tissues treated with osimertinib, the combination or control. A-C) Immunofluorescence images of DAPI, GFP, DPP4, NRF2 and CPT1A in lung tumor treated with osimertinib, the combination or control.

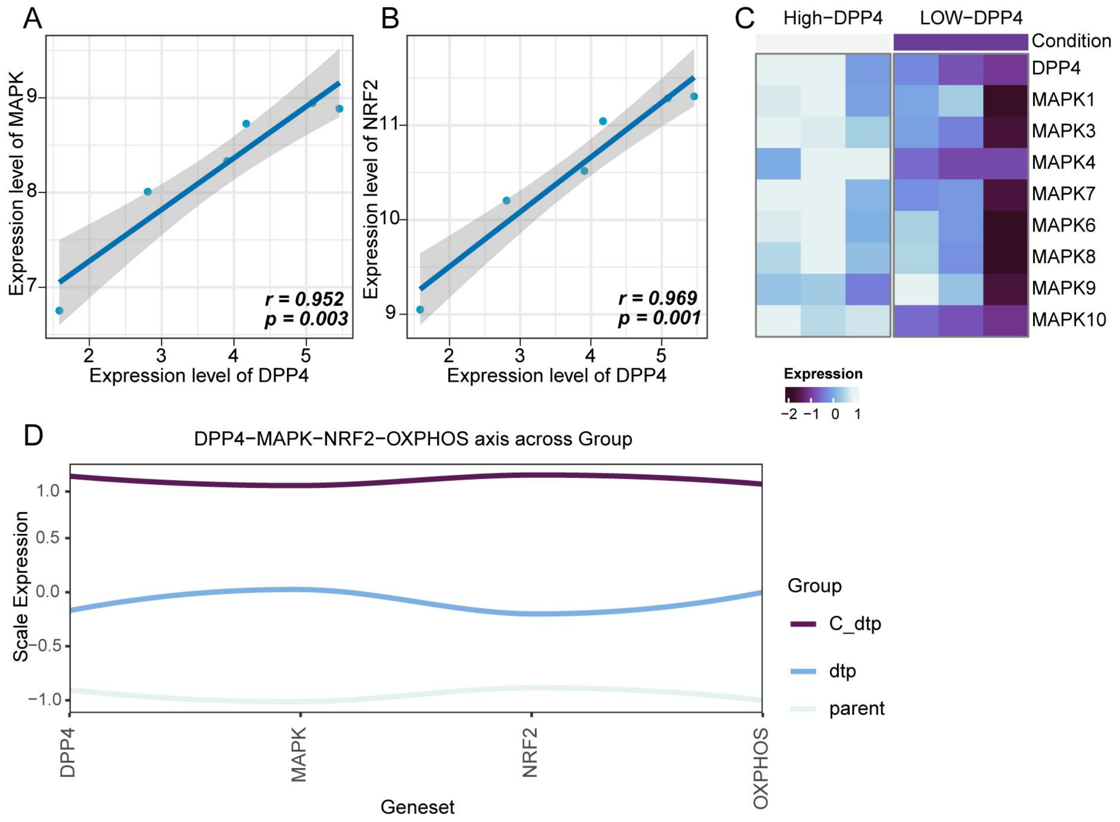


**Figure S17**

DPP4 maintains the DPP4-MAPK-NRF2-OXPHOS axis. A) Correlation analysis between DPP4 expression and the MAPK gene set expression levels (r = 0.952, p = 0.003). B) Correlation analysis between DPP4 expression and NRF2 expression levels (r = 0.969, p = 0.001). C) Heatmap showing expression of DPP4 and MAPK pathway genes in High-DPP4 and Low-DPP4 groups. Individual MAPK pathway genes (MAPK1, MAPK3, MAPK4, MAPK6, MAPK7, MAPK8, MAPK9, and

MAPK10) show consistently higher expression in the High-DPP4 group compared to the Low-DPP4 group. D) Scale expression analysis of the DPP4-MAPK-NRF2-OXPHOS axis across different cell groups. C_dtp cells (purple) maintain high expression across all pathway components, while dtp cells (blue) show moderate expression and parental cells (gray) exhibit minimal expression.

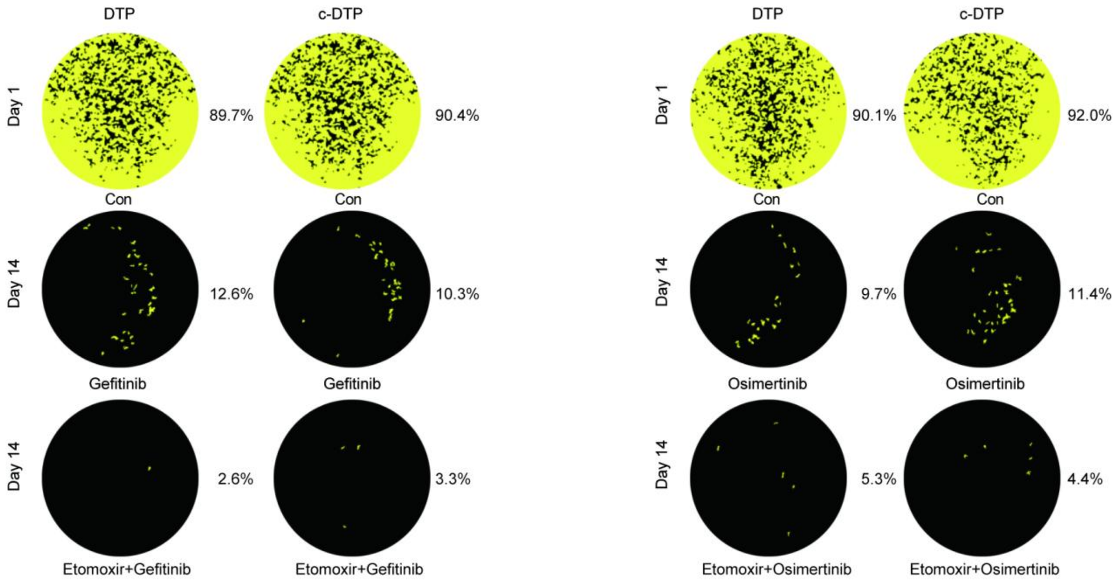


**Figure S18**

The number of cells after Etomoxir combined with gefitinib or osimertinib treatment was counted by Celigo Image Cytometer. Data are representative of three independent experiments.

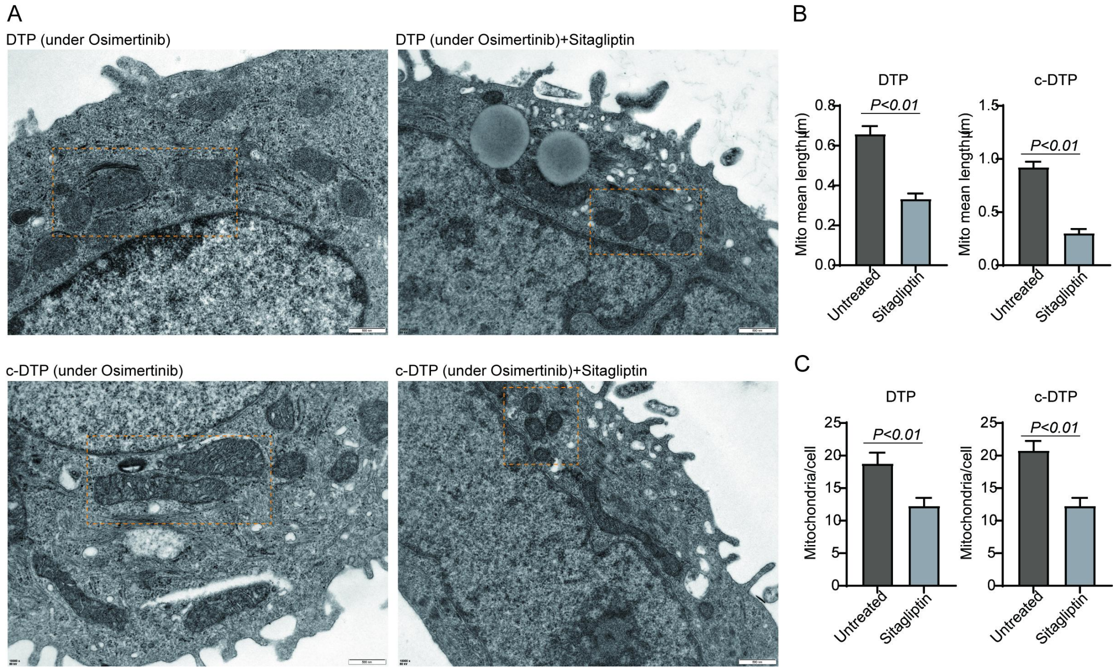


# FigureS19

A) Representative cell electron microscope results. The scale is 500nm.

B) Statistics of mitochondrial mean length. C) Statistics of average number of mitochondria.

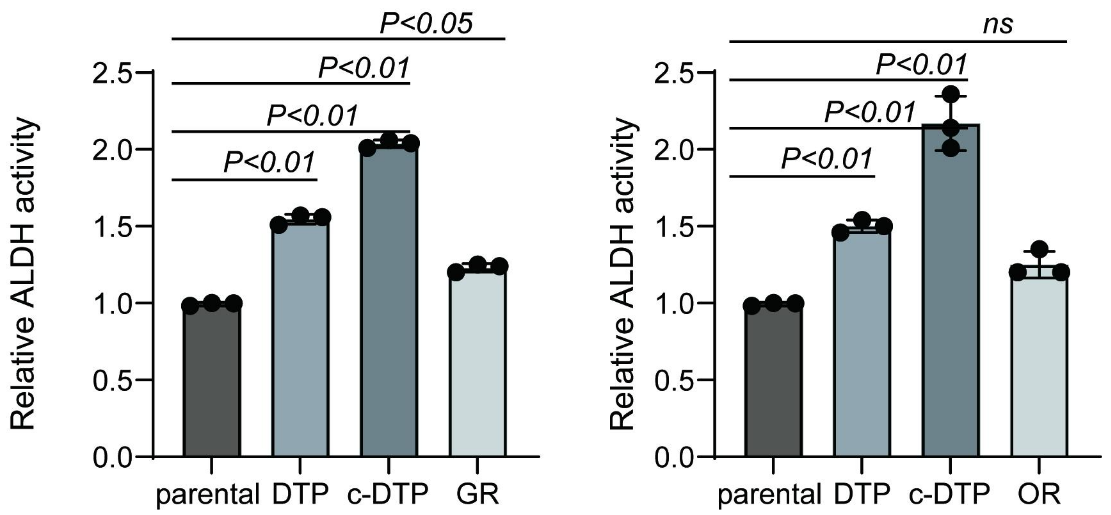


**Figure S20**

The activity of ALDH enzyme in parental cells, DTP cells, c-DTP cells and osimertinib /gefitinib-resistant cells. Higher OD values indicates stronger activity (n = 3).

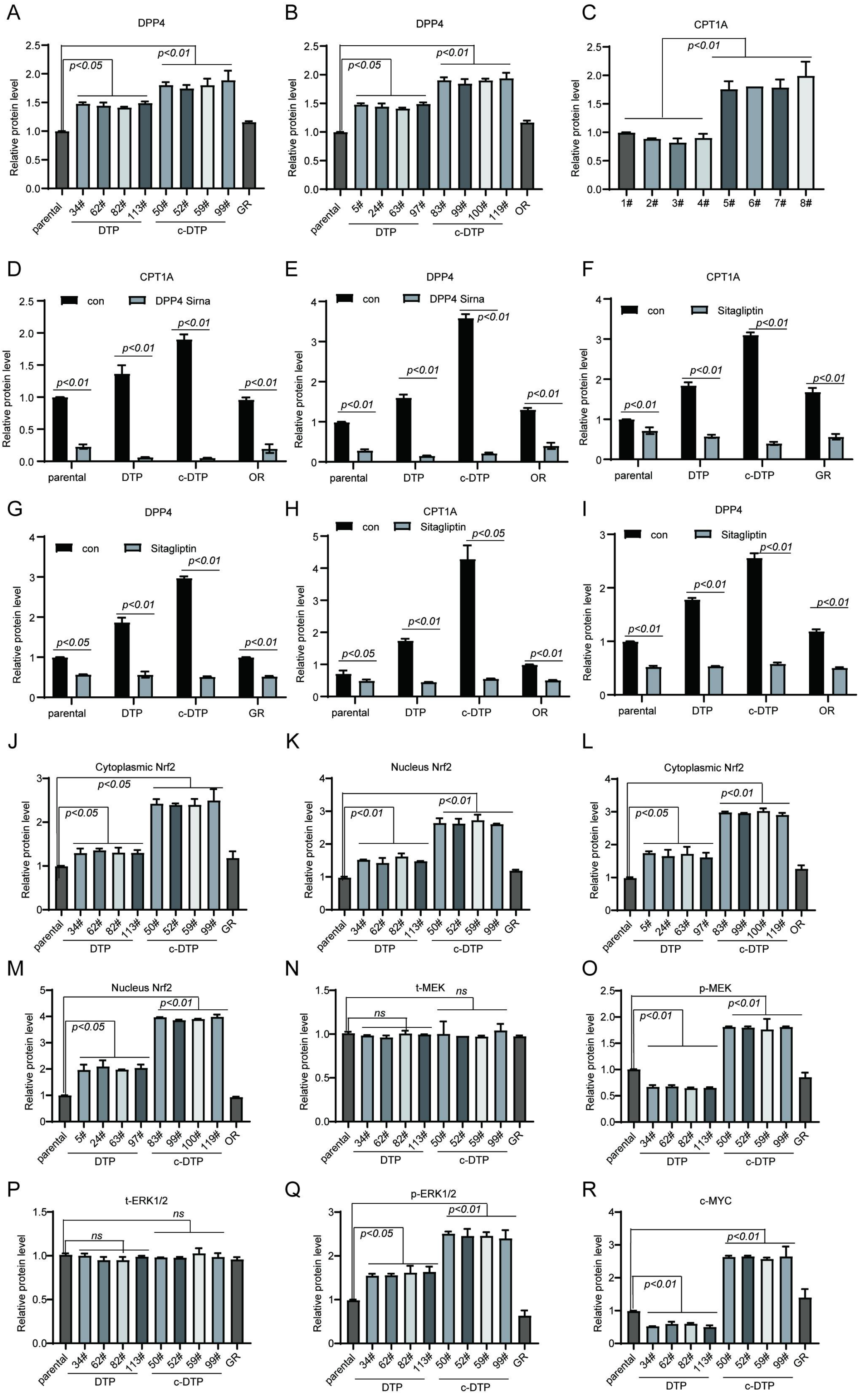
A
DPP4
B
DPP4
C
CPT1A
D
CPT1A
E
DPP4
F
CPT1A
G
DPP4
H
CPT1A
I
DPP4
J
Cytoplasmic Nrf2
K
Nucleus Nrf2
L
Cytoplasmic Nrf2
M
Nucleus Nrf2
N
t-MEK
O
p-MEK
P
t-ERK1/2
Q
p-ERK1/2
R
c-MYC
Relative protein level
parental
DTP
c-DTP
GR
OR
con
DPP4 Sirna
Sitagliptin
p<0.05
p<0.01
ns

## Figure S21

Quantitative analysis of Western blots. A) Quantitative analysis of Western blots in Figure 1D. B) Quantitative analysis of Western blots in Figure 1E. C) Quantitative analysis of Western blots in Figure 4F. D-E) Quantitative analysis of Western blots in Figure 4H. F-I) Quantitative analysis of Western blots in Figure 4I. J-M) Quantitative analysis of Western blots in Figure 5D. N-R) Quantitative analysis of Western blots in Figure 5-F.

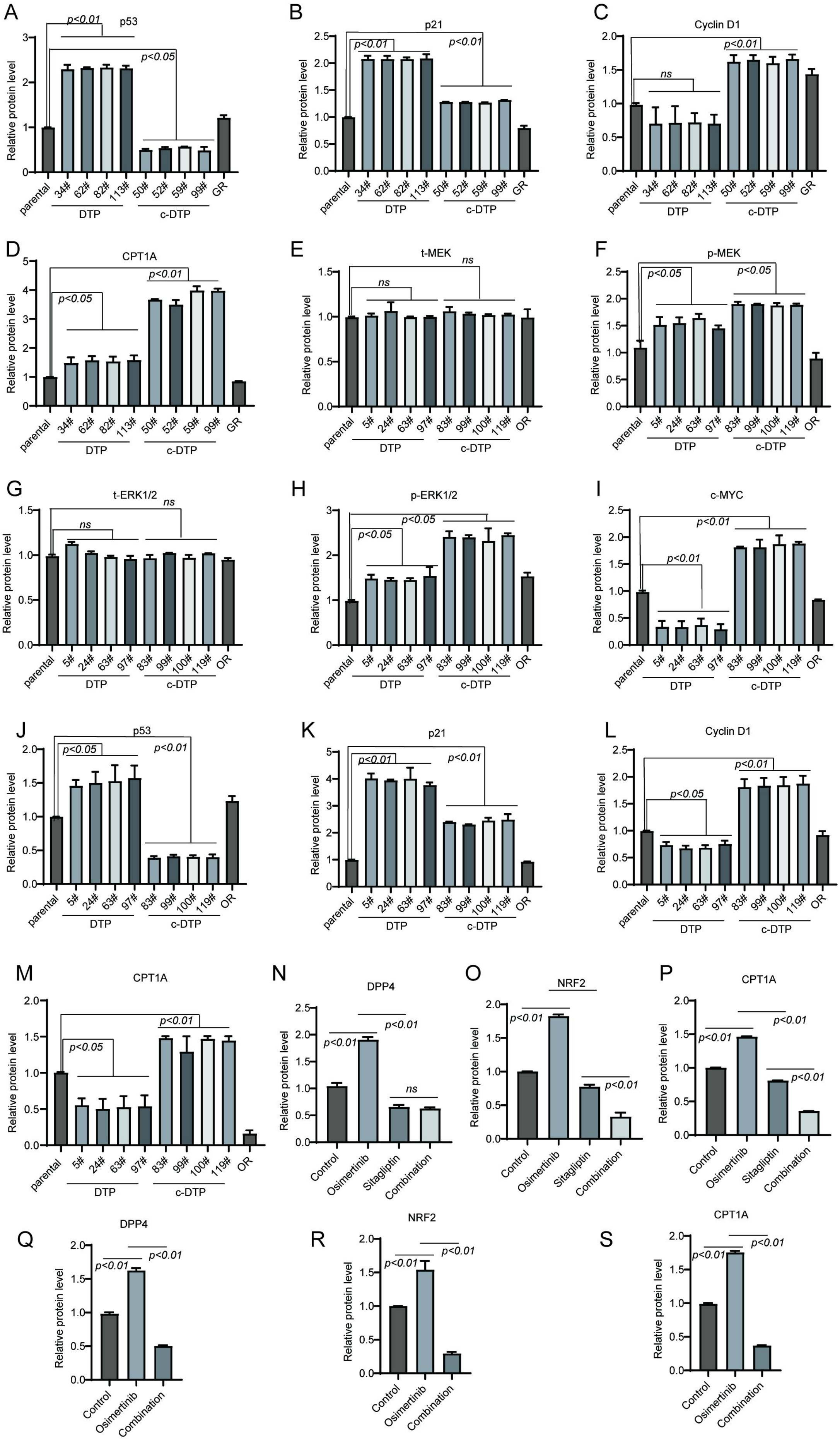

A
p53
p<0.01
p<0.05
Relative protein level
parental
34#
62#
82#
113#
50#
52#
59#
99#
GR
DTP
c-DTP
B
p21
p<0.01
p<0.01
C
Cyclin D1
ns
p<0.01
D
CPT1A
p<0.05
p<0.01
E
t-MEK
ns
ns
5#
24#
63#
97#
83#
99#
100#
119#
OR
F
p-MEK
p<0.05
p<0.05
G
t-ERK1/2
ns
ns
H
p-ERK1/2
p<0.05
p<0.05
I
c-MYC
p<0.01
p<0.01
J
p53
p<0.05
p<0.01
K
p21
p<0.01
p<0.01
L
Cyclin D1
p<0.05
p<0.01
M
CPT1A
p<0.05
p<0.01
N
DPP4
p<0.01
p<0.01
ns
Control
Osimertinib
Sitagliptin
Combination
O
NRF2
p<0.01
p<0.01
P
CPT1A
p<0.01
p<0.01
p<0.01
Q
DPP4
p<0.01
p<0.01
R
NRF2
p<0.01
p<0.01
S
CPT1A
p<0.01
p<0.01

## Figure S22

Quantitative analysis of Western blots. A-M) Quantitative analysis of Western blots in Figure 5F. N-P) Quantitative analysis of Western blots in Figure 6H. Q-S) Quantitative analysis of Western blots in Figure 7E.
